# Engineering Selenium–Chitosan Nanoparticles for Enhanced Hepatic Delivery of Sunitinib and Improved In Vitro Anticancer Activity in Hepatocellular Carcinoma Models

**DOI:** 10.3390/ph19060898

**Published:** 2026-06-05

**Authors:** Ahmed S.G. Srag El-Din, Eman Hamza, Ahmed Y. Kira, Sameh Saber, Mona H. Zohny, Ohoud Y. Alshehri, Reham A. Al-Dhelaan, Eslam Osama Mohamed, Heba I. Elagamy

**Affiliations:** 1 Department of Pharmaceutics, Faculty of Pharmacy, Delta University for Science and Technology, Gamasa 11152, Egypt; ahmed.kira@deltauniv.edu.eg (A.Y.K.); hebaelagamy1985@yahoo.com (H.I.E.); 2 Department of Biochemistry, College of Medicine, Imam Mohammad Ibn Saud Islamic University (IMSIU), Riyadh 13317, Saudi Arabia; oyaalshehri@imamu.edu.sa (O.Y.A.); raldhelaan@imamu.edu.sa (R.A.A.-D.); 3 Department of Pharmacology, Faculty of Pharmacy, Delta University for Science and Technology, Gamasa 11152, Egypt; sameh.saber@deltauniv.edu.eg; 4 Department of Biochemistry, Faculty of Pharmacy, Delta University for Science and Technology, Gamasa 11152, Egypt; muna.zohny@deltauniv.edu.eg; 5 Department of Biotechnology, Chemistry, and Pharmacy, University of Siena, 53100 Siena, Italy; e.osamaabdelkader@student.unisi.it

**Keywords:** sunitinib, selenium–chitosan nanoparticles, MDR-HepG2 cells, cytotoxicity, hepatic distribution, hepatocellular carcinoma

## Abstract

**Background/Objectives**: Hepatocellular carcinoma (HCC) remains difficult to treat because systemic therapy is constrained by limited selectivity, resistance, and toxicity. This study aimed to engineer selenium–chitosan nanoparticles loaded with sunitinib (SeNPs-Ch-SUN) to enhance hepatic delivery and improve anticancer activity against HCC. **Methods**: The developed system was characterized for particle size (PS), zeta potential (ZP), loading efficiency (LE%), in vitro release, and storage stability. Their cytotoxicity was evaluated in parental HepG2 and Huh-7 cells, multidrug-resistant HepG2 cells, SUN-resistant Huh-7 cells, and THLE-2 normal hepatocytes. In vivo hepatic distribution after intravenous administration was also assessed in rats. **Results**: SeNPs-Ch-SUN exhibited a mean PS of 93.62 ± 1.06 nm, positive ZP of +24.47 ± 1.31 mV, and LE of 83.8 ± 2.16%. FTIR supported drug association with the chitosan-stabilized selenium system. Compared with free sunitinib, SeNPs-Ch-SUN exhibited sustained drug release, with 51.17 ± 1.26% released at 24 h, whereas the free drug was almost completely released within 3 h. This controlled-release behavior translated in vivo into prolonged hepatic retention and superior liver exposure after intravenous administration. SeNPs-Ch-SUN significantly increased liver AUC_0–24_ to 77.23 ± 10.56 µg/g·h, compared with 36.39 ± 9.66 µg/g·h for free SUN, corresponding to an approximately 2.1-fold increase in hepatic exposure. SeNPs-Ch-SUN enhanced cytotoxicity in parental and resistant HCC models, lowered IC_50_ values, improved selectivity toward malignant cells, and reduced resistance index (RI) in MDR-HepG2 cells, while maintaining reduced toxicity toward normal hepatocytes relative to the free SUN. **Conclusions**: SeNPs-Ch-SUN represents a promising liver-directed nanoplatform for sunitinib delivery.

## 1. Introduction

Hepatocellular carcinoma (HCC) is the most prevalent type of primary liver cancer and remains one of the leading causes of cancer-related death worldwide [1]. Advances in imaging and liquid biopsy technologies have facilitated earlier detection, and treatment strategies are diversifying to include immune checkpoint inhibitors, tyrosine kinase inhibitors, and interventional therapies; however, the rising prevalence of HCC linked to non-infectious causes underscores the pressing demand for more effective diagnostic tools and therapeutic interventions [2]. According to GLOBOCAN 2022 data, there were approximately 684,659 new HCC cases and 597,434 deaths globally, with the highest age-standardized rates observed in Eastern Asia and Northern Africa. Overall, liver cancer, of which HCC constitutes the vast majority, represents the sixth most commonly diagnosed cancer and the third leading cause of cancer-related deaths worldwide. The 5-year survival rate for HCC remains approximately 18%, and despite a slowly decreasing trend in global age-standardized incidence rates since the late 1990s, the total number of HCC cases has been rising owing to aging and population growth. The geographic distribution of HCC is highly heterogeneous, with the highest incidence rates observed in East Asia and sub-Saharan Africa, a disparity largely driven by the endemicity of hepatitis B virus (HBV) and hepatitis C virus (HCV) in these regions [3,4].

The major risk factors for HCC include chronic HBV or HCV infection, aflatoxin exposure, heavy alcohol consumption, excess body weight, type 2 diabetes, and smoking, although the key determinants vary across regions. In most high-risk areas such as China and Eastern Africa, chronic HBV infection and aflatoxin exposure predominate, whereas HCV infection is the leading cause in countries such as Egypt, Italy, and Japan. In Western countries, the incidence of HCC has been rising, driven by increasing rates of alcohol abuse and metabolic dysfunction-associated steatotic liver disease (MASLD). Collectively, these diverse etiological pathways converge on chronic hepatic inflammation and fibrosis, ultimately culminating in malignant transformation [2,5].

Despite advances in surgery, local treatment, systemic therapy, and surveillance, most patients are still diagnosed with advanced disease, for which no curative options exist, and treatment outcomes are very poor [6,7]. Traditional treatments such as surgical resection and transarterial chemoembolization offer limited efficacy, especially in advanced stages. Although novel therapies like Lenvatinib, sorafenib, regorafenib, and immune checkpoint inhibitors (ICIs) have shown promise, their effectiveness is often hindered by primary and acquired resistance, leading to poor long-term survival outcomes. Sorafenib and Lenvatinib, the primary first-line targeted therapeutic drugs for HCC, face severe clinical limitations due to drug resistance, encompassing mechanisms such as non-coding RNA regulation, DNA methylation, and histone modification that drive aberrant signaling, metabolic reprogramming, and uncontrolled cell proliferation. Despite multiple first- and second-line regimens being approved for systemic treatment of HCC, the development of resistance and limitations of existing drug therapies highlight the urgent need for more precise drug selection and the development of agents with greater efficacy and improved safety profiles [8,9]. Therefore, systemic therapy is an important part of the management of advanced HCC, but its clinical effectiveness is limited by factors such as tumor heterogeneity, drug resistance, drug-related toxicity, and the liver function reserve of patients with cirrhosis [10,11].

Sunitinib (SUN) is a multi-target tyrosine kinase inhibitor that inhibits multiple signaling pathways critical for tumor progression and development [12]. These pathways include those mediated by the vascular endothelial growth factor receptors and those mediated by the platelet-derived growth factor receptors (i.e., their associated angiogenic activities) [13]. Since HCC is highly vascularized, SUN was considered an attractive biologic option for its treatment [14]. Unfortunately, therapeutic development has been limited by an unfavorable clinical profile: in a phase III study, SUN was shown to be inferior to the current standard of care for HCC, sorafenib, with respect to overall survival, and was associated with a greater incidence and severity of toxicity than sorafenib [15,16]. However, the described limitations do not negate the mechanistic rationale for using SUN in HCC; rather, they suggest additional efforts to optimize hepatic delivery and exposure.

Nanocarrier drug delivery systems offer a promising approach to reformulating anticancer drugs with limited clinical use by improving solubility, controlling release from the carrier, increasing cellular uptake, and possibly limiting off-target exposure [17,18]. In the case of HCC, this strategy is particularly relevant because drug distribution depends not only on tumor biology but also on the unique characteristics of the hepatic microenvironment [19,20,21]. Additionally, it is critical to preserve normal, non-cancerous hepatic tissues during drug therapy. Thus, by utilizing a rationally engineered nanoplatform, the pharmacological properties of SUN can be improved, and its interaction with HCC cells, particularly those with phenotypic characteristics of low drug sensitivity, can be enhanced.

Selenium nanoparticles (SeNPs) are one of the many desirable nanomaterials. They can act as both drug carriers and as active biologic agents [22]. Various selenium-based nanostructures have been found to induce oxidative stress imbalance, mitochondrial dysfunction, and apoptosis signaling in cancer cells, as well as provide an adaptable platform for drug loading [23]. SeNPs have been shown to reduce the expression of mTOR, AKT, and PI3K, as well as activate autophagy-related mediators, ultimately inducing cancer cell death through both apoptotic and autophagic pathways in liver cancer models. In the context of HCC specifically, several prior studies have demonstrated the therapeutic potential of SeNPs. Galactose-modified SeNPs loaded with doxorubicin (GA-Se@DOX) were engineered to target the asialoglycoprotein receptor highly expressed on HCC cells, demonstrating superior inhibition of HepG2 cell proliferation and apoptosis induction compared to free doxorubicin, both in vitro and in vivo, without observable damage to major organs [24]. Similarly, hyaluronic acid-functionalized SeNPs (HA-SeNPs) loaded with doxorubicin showed stronger ability to suppress HepG2 cell growth, migration, and invasion compared to either free doxorubicin or unmodified selenium nanoparticles, with cytotoxic activity mediated through ROS-induced apoptosis [25]. More recently, a biomimetic SeNP system encapsulated within tumor cell-derived extracellular vesicles (CCEVSe) demonstrated selective HCC targeting, achieving up to 87% tumor growth suppression in xenograft mouse models with Se concentrations in tumors approximately four times higher than free SeNPs, while exhibiting excellent safety with no observable systemic toxicity [26]. Additionally, berberine-loaded SeNPs exerted potent anticancer effects against HepG2 cells by inducing oxidative stress and activating apoptosis via upregulation of p53, Bax, and caspase-3, and downregulation of Bcl-2. These studies underscore the versatility of SeNPs as both cytotoxic agents and drug delivery vehicles in HCC [27].

The use of chitosan (Ch) for surface stabilization confers additional benefits, including colloidal stability, a positive surface charge, improved interaction with negatively charged cell membranes, and enhanced internalization [28,29]. For liver cancer, this combination of properties makes the SeNPs-Ch composite particularly attractive because it enables multiple mechanisms of action simultaneously: drug loading, sustained release, and biologically host-compatible nano-cell interactions. Chitosan-based NPs have been widely investigated as drug delivery systems because chitosan is a biodegradable and biocompatible cationic polysaccharide with abundant amino and hydroxyl groups that can participate in ionic interactions, hydrogen bonding, and surface stabilization [30]. These structural characteristics allow chitosan to act not only as a stabilizing polymer, but also as a functional interfacial layer that can improve NP dispersion, enhance drug association, and modulate drug release. In hepatic drug delivery, chitosan-based NPs improved liver-directed delivery and potentiated anticancer activity in vitro and in a N-nitrosodiethylamine-induced HCC mouse model [19,31].

Building on this concept, we developed the SeNPs-Ch nanoplatform to optimize hepatic delivery of SUN for the treatment of HCC. The prepared NPs were characterized regarding the physical characteristics: particle size distribution, zeta potential (ZP), loading efficiency (LE), in vitro release profile, formulation stability, and in vitro anticancer activity against HCC, including both sensitive and resistant phenotypes. An in vivo hepatic distribution study was also conducted to determine whether the formulation would enhance hepatic delivery compared with free SUN. Thus, it is hypothesized that incorporating SUN into SeNPs-Ch would improve SUN’s delivery profile and efficacy, enhance SUN’s cellular anticancer effects, and promote preferential hepatic accumulation of SUN, all of which support the potential of this nanostructured therapeutic approach for HCC.

## 2. Results and Discussion

### 2.1. Characterization of SeNPs-Ch-SUN

**Figure 1:**
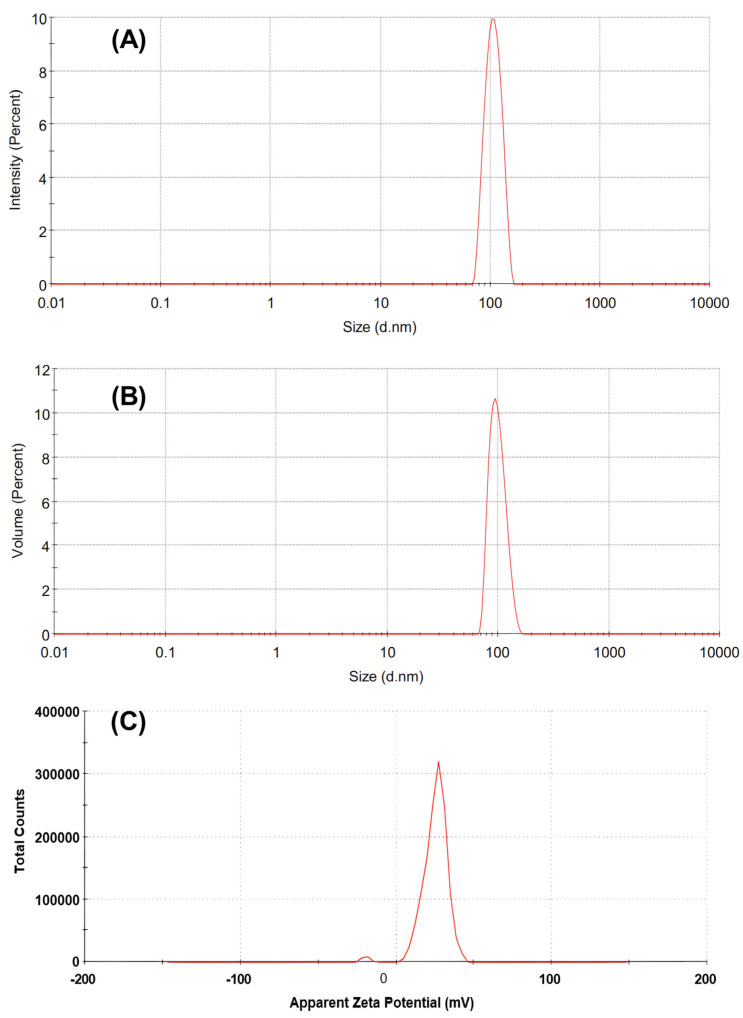
(**A**) Intensity-based, (**B**) volume-based size distribution measured by DLS, and (**C**) Zeta-potential distribution of SeNPs-Ch-SUN.

SeNPs-Ch-SUN was successfully fabricated as a homogeneous orange-red colloidal dispersion without visible macroscopic phase separation or precipitation (Supplementary Figure S1). The prepared SeNPs-Ch-SUN exhibited a mean PS of 93.62 ± 1.06 nm, as shown in Figure 1A,B, representing both intensity- and volume-based size-distribution profiles, respectively. PS is an important parameter for hepatic delivery, as previous studies have shown that liver sinusoidal endothelial fenestrations are typically 150–200 nm in diameter [32,33]. Thus, PS < 200 nm is likely able to cross liver sinusoidal fenestrations and access the space of Disse. In addition, the PS value obtained in our study is well above the estimated renal filtration limit of approximately 5.5 nm, making rapid renal excretion unlikely after administration [34]. The PDI of SeNPs-Ch-SUN was 0.205 ± 0.01, indicating a homogeneous size distribution, as values below 0.3 are generally considered indicative of a narrow and acceptable size distribution [35].

Regarding surface charge, SeNPs-Ch-SUN exhibited a positive ZP of +24.47 ± 1.31 mV (Figure 1C). The positive charge obtained is consistent with the presence of Ch on the particle surface, as protonated NH_3_ groups provide cationic character to the SeNPs. The positively charged NPs obtained can improve the cellular delivery of SUN, as cationic NPs interact more readily with negatively charged cellular membranes, thereby favoring cell association and internalization [36].

Regarding the LE%, SeNPs-Ch-SUN exhibited a value of 83.8 ± 2.16%, indicating efficient association of SUN with the SeNPs-Ch. The distinct physicochemical features of the prepared NPs were shown to be responsible for the exceptionally high drug LE. The high surface-to-volume ratio of SeNPs-Ch-SUN and the functional groups of Ch provide many anchoring sites for SUN molecules to bind effectively, as supported by the FTIR study.

### 2.2. FTIR Analysis and Morphological Evaluation of SeNPs-Ch-SUN

**Figure 2:**
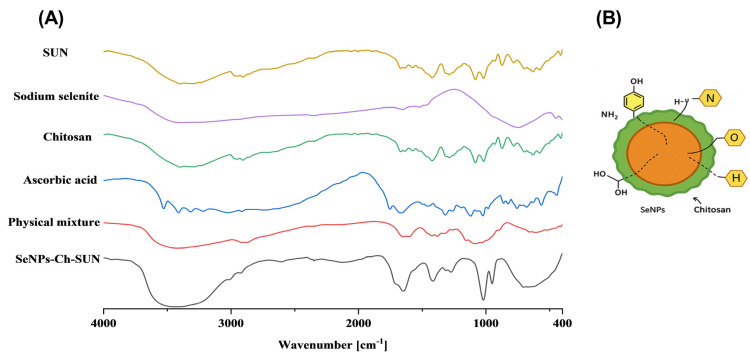
(**A**) FTIR spectra of SUN, chitosan, sodium selenite, ascorbic acid, their physical mixture, and SeNPs-Ch-SUN. (**B**) Schematic illustration of the proposed interactions within SeNPs-Ch-SUN, showing a selenium core stabilized by chitosan and associated with SUN through plausible hydrogen-bonding and electrostatic interactions.

The FTIR spectra of pure SUN, sodium selenite, chitosan, ascorbic acid, their physical mixture, and SeNPs-Ch-SUN are shown in Figure 2A. The FTIR spectrum of SUN exhibited a broad absorption peak around 3340 cm^−1^, which is attributed to the N–H stretching vibrations of secondary amines. Additionally, a slight peak observed around 2940 cm^−1^ is likely due to the CH_2_ asymmetric stretching of the aliphatic side chains. A moderate peak at 1424 cm^−1^ is typically associated with C-H bending vibrations. The peak at 1018 cm^−1^ corresponds to the C–N stretching vibrations within the aliphatic amine moieties of the SUN compound [37]. The FTIR spectrum of Sodium Selenite displayed a prominent peak at approximately 740 cm^−1^, indicative of the O–Se–O bending vibration (rocking mode) characteristic of the selenite ion. This bending vibration is a defining feature of the selenite group, confirming the structural integrity of the SeO_3_^2−^ species within sodium selenite and indicating that the compound exists in its expected anionic form without undergoing reduction or structural degradation [38].

The FTIR spectrum of chitosan revealed a broad band at 3421 cm^−1^, corresponding to O–H and N–H stretching. A peak at 1650 cm^−1^ corresponds to C=O stretching, attributed to amide. Additionally, the peak at 1070 cm^−1^ is associated with C–O–C stretching, which is representative of the polysaccharide backbone [39]. The FTIR spectrum of ascorbic acid exhibited strong bands at 3400 cm^−1^ (O–H stretching), 1750 cm^−1^ (C=O stretching in the lactone ring), 1650 cm^−1^ (C=C stretching), and 1050 cm^−1^ (C–O stretching) [40].

The FTIR spectrum of the physical mixture of the components displayed all the characteristic peaks of the individual substances without significant shifts or disappearance, indicating that no interactions occurred between the components. This suggests that the components retain their chemical integrity and that the interactions in the mixture are primarily physical rather than chemical. In the FTIR spectrum of the SeNPs-Ch-SUN, the broad peak at 3426 cm^−1^ signifies a chemical interaction between the N–H stretching of SUN (3340 cm^−1^) with OH and NH groups of chitosan that coat the SeNPs-Ch-SUN [41]. The characteristics of the FTIR bands of the individual components, physical mixture, and SeNPs-Ch-SUN, with the main spectral changes supporting drug association, chitosan surface stabilization, and SeNP formation, are summarized in Supplementary Table S1.

The interaction model represented in the schematic (Figure 2B) is interpreted as a potential mechanism for how the two components interact, rather than an actual mechanistic proof. Based on the FTIR studies, it is likely that the Se core is stabilized by Ch, that SUN is associated with the selenium-core/chitosan interfaces through hydrogen-bonding and electrostatic interactions, and that this arrangement accounts for the relatively high LE% observed for these formulations; thus, it is a reasonable explanation for the sustained-release characteristics discussed in the release study later. By allowing association of the drug without requiring irreversible alterations to the physical or chemical structure of SUN, the SeNPs-Ch system may continue to enable improved drug distribution to the liver and maintain the drug’s pharmacological availability.

**Figure 3:**
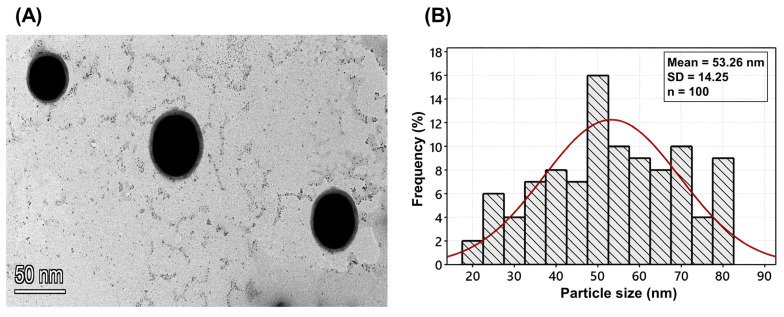
TEM morphology and particle size distribution of SeNPs-Ch-SUN. (**A**) Representative TEM micrograph showing discrete, nearly spherical SeNPs-Ch-SUN nanoparticles at the nanoscale. (**B**) TEM-based particle size distribution histogram obtained from the measurement of 100 individual nanoparticles, showing a mean particle diameter of 53.26 ± 14.25 nm.

The TEM micrograph (Figure 3A) showed that SeNPs-Ch-SUN consisted of discrete, nearly spherical nanoparticles with no obvious large-scale aggregation. To provide quantitative morphological size analysis, the diameters of 100 randomly selected particles were measured from the TEM image using ImageJ software Version 1.54d and plotted as a particle-size distribution histogram (Figure 3B). The mean TEM particle diameter was 53.26 ± 14.25 nm (*n* = 100), confirming the formation of a nanosized system. This value was lower than the hydrodynamic diameter obtained by DLS (93.62 ± 1.06 nm), which is consistent with previous reports [42,43]. The relatively lower values obtained by TEM compared with DLS can be attributed to differences between the techniques. TEM measures the dried physical particle size, whereas DLS reflects the hydrodynamic diameter of particles in dispersion, including the solvated polymer layer. The absence of marked aggregation in the TEM image is consistent with the relatively low PDI observed for the formulation, and supports the stabilizing role of chitosan at the nanoparticle surface.

### 2.3. In Vitro Release and Release Kinetics

**Figure 4:**
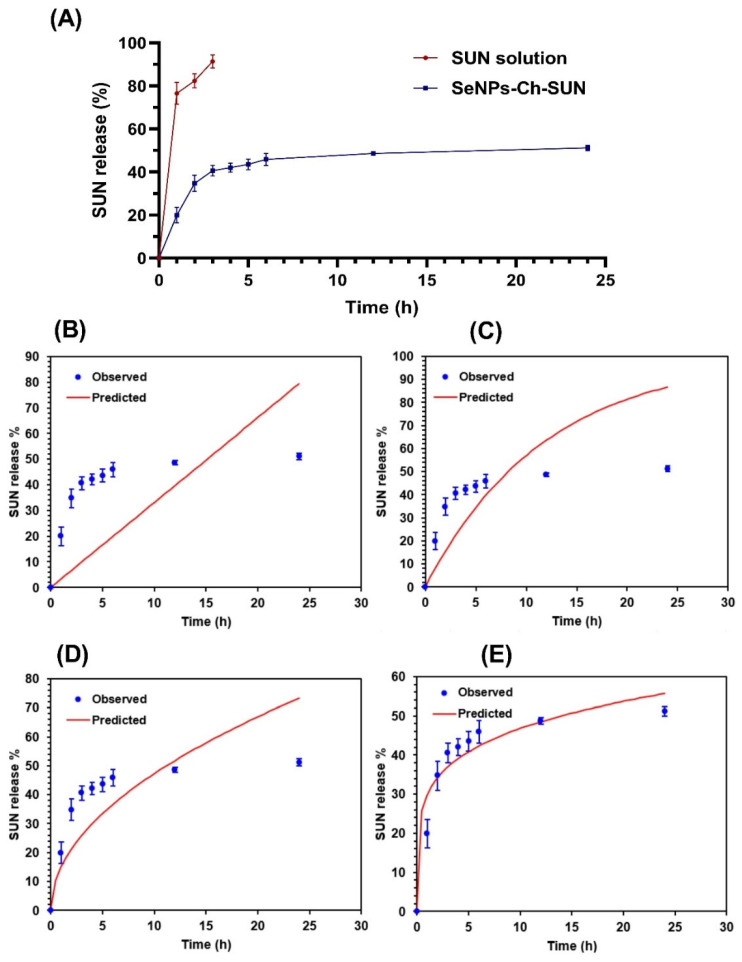
In vitro release profile and release-kinetic analysis of SUN from SeNPs-Ch-SUN. (**A**) Comparative release profiles of free SUN solution and SeNPs-Ch-SUN in PBS (pH 7.4) for 24 h. Data are presented as mean ± SD, *n* = 3. (**B**) Zero-order kinetic plot. (**C**) First-order kinetic plot. (**D**) Higuchi kinetic plot. (**E**) Korsmeyer–Peppas kinetic plot. The blue dots represent the experimentally observed release data, whereas the red line represents the release profile predicted by the corresponding kinetic model. The Korsmeyer–Peppas model showed the best fit to the release data, indicating diffusion-dominated release from the nanoparticle system.

The in vitro release profiles of free SUN and SeNPs-Ch-SUN are presented in Figure 4A. Free SUN showed rapid release, with 76.56 ± 5.11% released within 1 h and nearly 100% at 3 h. This burst release demonstrates the rapid dissolution of free SUN in the release medium, with a significantly higher percentage than that of SeNPs (*p* < 0.05). In contrast, SeNPs-Ch-SUN exhibited a distinctly slower release pattern, with 19.92 ± 3.60% released at 1 h. Then, the release% of SeNPs-Ch-SUN increased gradually in a sustained manner, reaching 51.17 ± 1.26% at 24 h. The sustained-release characteristic can be attributed to the diffusion of encapsulated drug molecules from the SeNP core through the chitosan coating, which is controlled by both electrostatic and hydrogen-bonding interactions between SUN and the polymer matrix [44,45]. This behavior may also reflect the presence of a strongly associated SUN fraction retained within the NP matrix or at the Se/Ch interfacial region. In this system, the chitosan coating likely acts as a diffusional barrier that slows the outward movement of SUN from the NP matrix into the external release medium. In addition, non-covalent interactions between SUN and chitosan functional groups, including hydrogen bonding and electrostatic interactions suggested by the FTIR findings, may further restrict rapid drug diffusion. Therefore, the approximately 50% release after 24 h reflects sustained and diffusion-controlled release rather than incomplete formulation performance.

These results indicate that incorporating SUN into SeNPs-Ch markedly slowed drug release compared with the free drug solution. Rapid release of free SUN was expected, as the drug was in a freely diffusible form with no carrier-related barrier to dissolution and diffusion. By contrast, SeNPs-Ch-SUN exhibited a biphasic profile, with an initial rapid phase followed by a slower, more sustained phase. The initial phase most likely reflects the release of the fraction of SUN weakly associated with or located near the outer chitosan-rich interfacial region, whereas the subsequent slower phase can be attributed to the gradual diffusion of more strongly associated drug from the nanoparticle system. The slower release rate of the developed SeNPs-Ch may help limit the abrupt drug availability observed with the free solution, reducing premature release into the systemic circulation and enhancing drug residence in hepatic tissues.

To further characterize the release mechanism, the SeNPs-Ch-SUN release data were fitted to zero-order, first-order, Higuchi, and Korsmeyer–Peppas models (Figure 4B–E). Among the tested models, the Korsmeyer–Peppas model provided the best fit, with R^2^ values of 0.925 ± 0.012. In comparison, the Higuchi model showed only moderate fitting, whereas the zero-order and first-order models provided poorer descriptions of the observed release behavior. The release exponent obtained from the Korsmeyer–Peppas model was *n* = 0.198 ± 0.042, which is <0.45, indicating a diffusion-dominated release. Thus, the release of SUN from SeNPs-Ch-SUN appears to be governed primarily by Fickian diffusion through the nanoparticle-associated matrix/interface rather than by erosion-controlled release.

### 2.4. Stability Study

**Figure 5:**
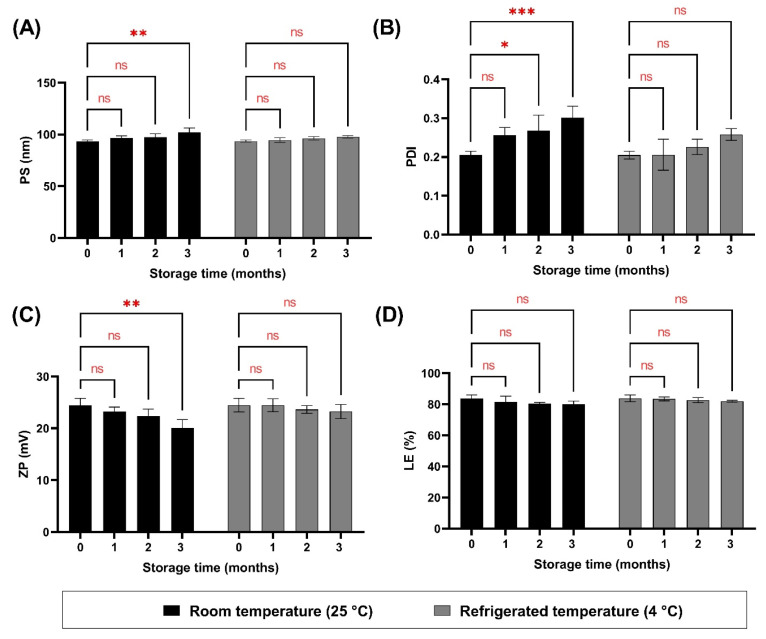
Stability profile of SeNPs-Ch-SUN during storage for 3 months at room temperature (25 °C) and refrigerated temperature (4 °C). Changes in (**A**) particle size (PS), (**B**) polydispersity index (PDI), (**C**) zeta potential (ZP), and (**D**) loading efficiency (LE%) were monitored at 0, 1, 2, and 3 months. Data are presented as mean ± SD (*n* = 3). Statistical significance is indicated as follows: * *p* < 0.05, ** *p* < 0.01, *** *p* < 0.001, while ns indicates non-significant differences.

The storage stability of SeNPs-Ch-SUN was evaluated over 3 months under refrigerated (4 °C) and room-temperature (25 °C) conditions by monitoring PS, PDI, ZP, and LE% (Figure 5A–D). As shown in Figure 5A, during storage at 25 °C, PS exhibited a gradual increase that became statistically significant at the final time point, likely reflecting progressive interparticle association rather than abrupt aggregation. By contrast, under 4 °C, PS remained essentially unchanged throughout the study, with no significant deviation from the initial value. This behavior indicates that refrigerated storage was more effective at preserving the formulation’s original nanoscale dimensions. A similar trend was observed for PDI (Figure 5B). Thus, the refrigerated condition better preserved the monodisperse nature of SeNPs-Ch-SUN.

The ZP results further supported the superior stability of the refrigerated formulation (Figure 5C). During storage at 25 °C, ZP gradually declined, with a significant reduction at the final time point. This decrease suggests partial weakening of the electrostatic stabilization of the colloidal system. In contrast, only minor, non-significant fluctuations in ZP were observed at 4 °C, indicating that the formulation’s cationic surface characteristics were largely preserved under refrigerated conditions. Since electrostatic repulsion contributes to resistance against particle aggregation, the observed reduction in ZP at room temperature is consistent with the simultaneous increase in PS and PDI under the same conditions.

In contrast to the storage-dependent changes in colloidal parameters, LE% remained essentially unchanged under both conditions throughout the study (Figure 5D). No statistically significant differences were observed relative to the initial value at either 25 °C or 4 °C, indicating that the association of SUN with the SeNPs-Ch was maintained during storage. Based on these findings, refrigerated conditions appear to be the preferred storage condition for the developed formulation.

### 2.5. In Vitro Cytotoxicity

**Figure 6:**
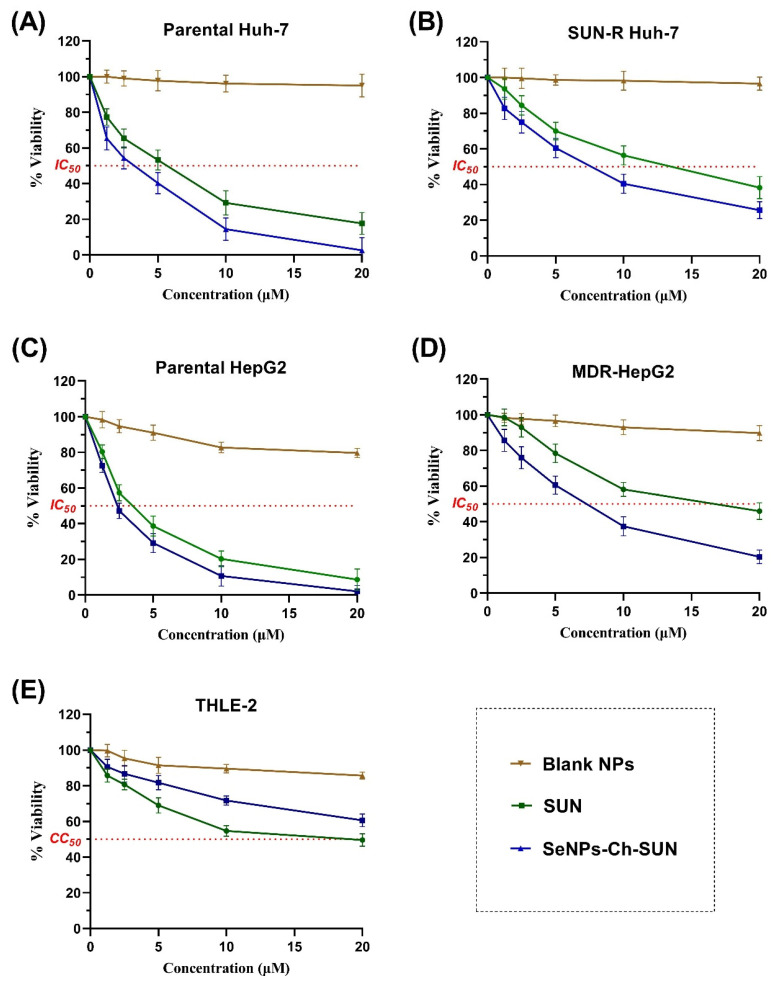
In vitro cytotoxicity of free SUN, blank SeNPs-Ch, and SeNPs-Ch-SUN after 48 h exposure in normal and HCC cell models. (**A**) parental Huh-7 cells, (**B**) SUN-resistant Huh-7 cells, (**C**) parental HepG2 cells, (**D**) MDR-HepG2 cells, and (**E**) THLE-2 normal human hepatocytes. Cell viability was determined by the MTT assay and expressed as a percentage relative to untreated control cells. Data are presented as mean ± SD (*n* = 3). The red dashed horizontal line indicates 50% viability (IC_50_ threshold). Blank SeNPs-Ch was tested at carrier-equivalent concentrations corresponding to those present in the respective SeNPs-Ch-SUN doses.

The in vitro cytotoxic activity of free SUN, blank SeNPs-Ch, and SeNPs-Ch-SUN was evaluated in parental and resistant HCC cell models, along with THLE-2 normal hepatocytes, after 48 h of exposure (Figure 6). Across all malignant cell models, SeNPs-Ch-SUN reduced cell viability more than free SUN across the tested concentration range, whereas blank SeNPs-Ch showed only limited cytotoxicity. In contrast, THLE-2 cells were less affected by SeNPs-Ch-SUN than by free SUN, indicating improved selectivity of the nanoformulation for malignant hepatic cells. This pattern was further supported by the calculated IC_50_*, RI*, and *SI* values summarized in Table 1.

In parental Huh-7 cells (Figure 6A), both free SUN and SeNPs-Ch-SUN reduced cell viability in a concentration-dependent manner; however, the SeNPs-Ch-SUN consistently exerted stronger cytotoxicity at matched concentrations. At 20 µM, SeNPs-Ch-SUN reduced viability to 2.62 ± 7.10%, compared with 17.62 ± 6.10% for free SUN, while blank SeNPs-Ch maintained viability at 94.99 ± 6.37%. This pattern was reflected in the IC_50_ values, which decreased significantly (*p* < 0.05) from 4.755 ± 0.707 µM for free SUN to 2.613 ± 0.575 µM for SeNPs-Ch-SUN. A similar trend was observed in parental HepG2 cells (Figure 6C), where SeNPs-Ch-SUN also produced greater viability loss than free SUN across the tested range, with IC_50_ values of 2.009 ± 0.485 µM and 3.317 ± 0.566 µM, respectively. These results indicate that SeNPs-Ch enhanced the in vitro cytotoxic potency of SUN in both HCC cell lines. These findings align with previous studies demonstrating that drug-loaded nanocarriers consistently exhibit greater cytotoxicity than equivalent concentrations of free drugs [46,47].

In the SUN-resistant Huh-7 model (Figure 6B), SeNPs-Ch-SUN again showed greater cytotoxicity than free SUN across all tested concentrations. At 20 µM, viability decreased to 25.60 ± 4.78% with SeNPs-Ch-SUN, compared with 38.25 ± 6.20% for free SUN. The corresponding IC_50_ values were 7.121 ± 0.959 µM for SeNPs-Ch-SUN and 12.747 ± 1.817 µM for free SUN, confirming improved potency of the SeNPs-Ch-SUN. However, when resistance was normalized against the parental Huh-7 line, the *RI* remained comparable for free SUN (2.68 ± 0.01) and SeNPs-Ch-SUN (2.76 ± 0.24). Thus, the prepared NPs clearly improved absolute cytotoxic potency but did not significantly reduce the relative magnitude of resistance. This observation aligns with reports demonstrating that nanoencapsulation improves absolute cytotoxic potency by rerouting drug internalization through endocytosis, thereby bypassing efflux-mediated clearance equally in both parental and resistant cells, resulting in a proportional IC_50_ reduction across both lines without altering the fold-resistance ratio [48]. Thus, as acquired SUN resistance may persist through intracellular adaptive mechanisms beyond drug access alone, SeNPs-Ch can improve potency without necessarily resulting in a marked reduction in the resistance index.

A different pattern was observed in MDR-HepG2 cells (Figure 6D). In this model, SeNPs-Ch-SUN produced a significant improvement in cytotoxic activity relative to free SUN, reducing viability to 20.36 ± 3.80% at 20 µM, compared with 45.95 ± 4.70% for free SUN. The IC_50_ decreased significantly (*p* < 0.05) from 17.557 ± 3.096 µM for free SUN to 6.787 ± 1.070 µM for SeNPs-Ch-SUN. These effects align with prior reports that nanoformulations can enhance the delivery and cytotoxicity of kinase inhibitors in HCC contexts [49]. Importantly, the *RI* was significantly reduced from 5.29 for free SUN to 3.38 for SeNPs-Ch-SUN, indicating that the SeNPs-Ch attenuated the MDR-associated loss of sensitivity in HepG2 cells more effectively than it did in the SUN-resistant Huh-7 model. This differential pattern may be attributable, at least in part, to the mechanistic nature of resistance in MDR-HepG2 cells, where P-glycoprotein (P-gp/ABCB1) overexpression is a dominant efflux-mediated resistance driver amenable to nanocarrier-based circumvention via endocytic uptake, thereby shielding encapsulated SUN from P-gp recognition and expulsion [50]. Chitosan itself has a documented capacity to suppress P-gp activity through inhibition of ATP synthesis and disruption of mitochondrial membrane potential, providing an additional contribution beyond passive delivery enhancement in P-gp-overexpressing MDR models [51].

Evaluation in THLE-2 normal hepatocytes (Figure 6E) further supported the formulation’s cytotoxic profile. Free SUN showed higher toxicity toward THLE-2 cells than SeNPs-Ch-SUN, with IC_50_ values of 13.567 ± 2.696 µM and >20 µM, respectively. This translated into substantial improvement in selectivity indices for the SeNPs-Ch-SUN. Compared with free SUN, SeNPs-Ch-SUN significantly increased the *SI* in parental HepG2 cells, indicating that the SeNPs-Ch not only enhanced cytotoxic potency in malignant hepatic cells, but also improved specificity toward cancerous tissues and avoided non-malignant hepatocytes. This selectivity pattern is in line with previous studies showing that free sunitinib can be cytotoxic to non-malignant cells, including normal hepatocytes, whereas nanocarrier-based sunitinib delivery may preserve antitumor activity while reducing toxicity toward normal cells [52,53].

Regarding Blank formulation, it produced minimal reductions in viability in Huh-7, SUN-resistant Huh-7, MDR-HepG2, and THLE-2 cells, although a more noticeable (though still partial) effect was observed in parental HepG2 cells at the highest tested concentration. These findings indicate that the carrier itself contributes some biological activity. This limited carrier-associated antiproliferative effect is consistent with previous reports showing that selenium/chitosan-based systems can themselves exert measurable inhibitory activity against HepG2 cells [54,55]. However, the markedly greater cytotoxicity of SeNPs-Ch-SUN compared with blank SeNPs-Ch shows that the effect of the loaded formulation cannot be attributed to the carrier alone. Rather, the enhanced activity of SeNPs-Ch-SUN most likely reflects a combined contribution of carrier-associated biological activity and improved cellular delivery of SUN.

Pairwise statistical comparison at matched concentrations further supported these observations (Supplementary Figure S2). SeNPs-Ch-SUN produced significantly greater viability reduction than free SUN in multiple HCC models, particularly in parental Huh-7, parental HepG2, and MDR-HepG2 cells, while blank SeNPs-Ch showed significantly weaker cytotoxicity than SeNPs-Ch-SUN at most tested concentrations. In THLE-2 normal hepatocytes, SeNPs-Ch-SUN maintained higher cell viability than free SUN at several concentrations, supporting the improved selectivity profile of the nanoformulation.

Compared with earlier reports, SeNPs-Ch-SUN provides the combined advantages of carrier-associated bioactivity, enhanced SUN potency, improved selectivity, and partial reversal of MDR-associated resistance. Sun et al. reported that Se-modified chitosan inhibited HepG2 cell proliferation across a broad concentration range and induced apoptosis through S/G_2_-M cell-cycle arrest, mitochondrial membrane potential disruption, and cleaved caspase-3 [56]. In the present study, blank SeNPs-Ch showed only limited cytotoxicity within the tested carrier-equivalent range, whereas SeNPs-Ch-SUN markedly enhanced SUN cytotoxicity, indicating that the biological response was not attributable to the carrier alone.

The improved cytotoxic profile is also consistent with previous studies of SUN nanocarriers. Dehghan et al. reported that SUN-loaded niosomes produced greater in vitro anticancer activity compared with free SUN [47]. Similarly, Yongvongsoontorn et al. showed that SUN-loaded PEG-EGCG micellar nanocomplexes enhanced anticancer activity and reduced toxicity compared with free SUN in tumor-bearing models, supporting the concept that carrier design can improve the therapeutic behavior of SUN beyond simple solubilization [57]. Importantly, the present study extends this concept to HCC-relevant parental and resistant hepatic cancer models and demonstrates that SeNPs-Ch-SUN not only enhanced cytotoxicity but also improved selectivity relative to THLE-2 normal hepatocytes.

### 2.6. In Vivo Hepatic Targeting

**Figure 7:**
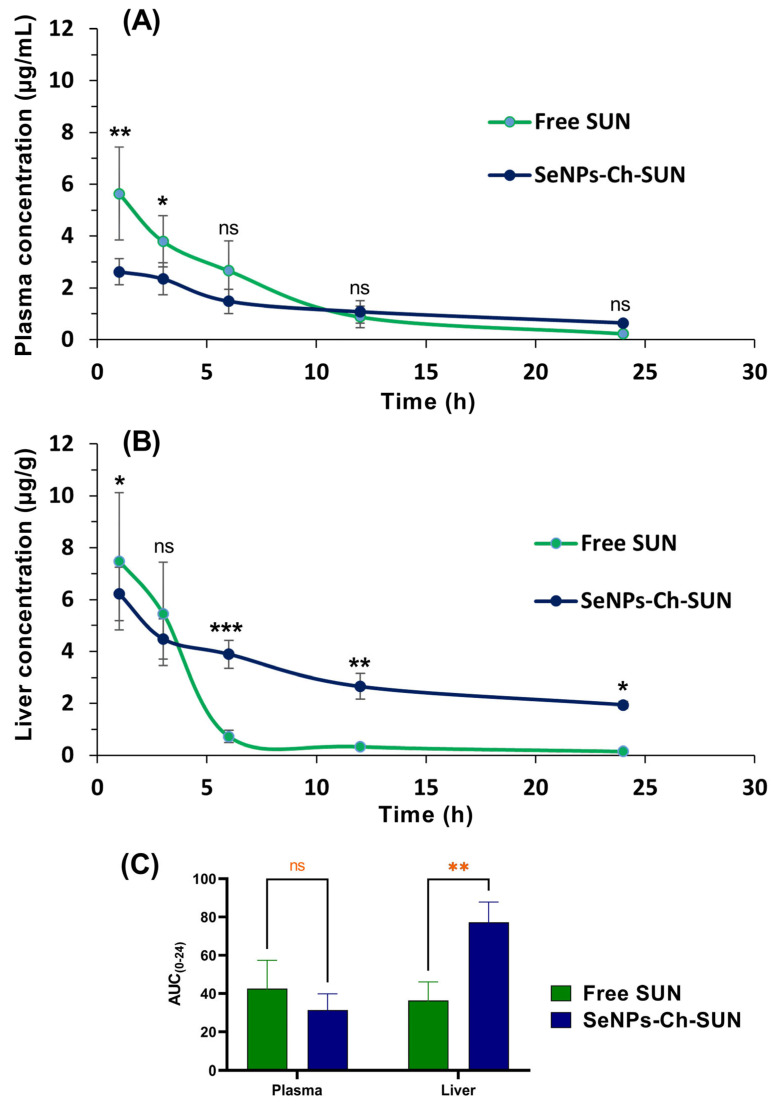
In vivo hepatic distribution profile of free SUN and SeNPs-Ch-SUN following a single IV dose equivalent to 5 mg/kg in rats. (**A**) Plasma concentration–time profile. (**B**) Liver concentration–time profile. (**C**) Comparative plasma and liver exposure expressed as AUC_0-24._ Data are presented as mean ± SD (*n* = 3). Statistical comparisons were performed between free SUN and SeNPs-Ch-SUN at each corresponding time point or tissue compartment. Significance is indicated as follows: ns, non-significant; * *p* < 0.05; ** *p* < 0.01; *** *p* < 0.001.

The plasma and liver concentration–time profiles of free SUN and SeNPs-Ch-SUN following a single IV dose equivalent to 5 mg/kg are shown in Figure 7A,B. In plasma, free SUN exhibited a higher initial concentration at 1 h (5.64 ± 1.79 µg/mL) than SeNPs-Ch-SUN (2.62 ± 0.51 µg/mL). However, SeNPs-Ch-SUN maintained measurable concentrations across the study interval. At 24 h, the plasma concentration of SeNPs-Ch-SUN (0.64 ± 0.07 µg/mL) remained higher than that of free SUN (0.23 ± 0.09 µg/mL), indicating more prolonged systemic persistence of the nanoparticle-associated drug. This behavior corresponds to the sustained-release profile observed with SeNPs-Ch-SUN in vitro, indicating that a large portion of the drug remained associated with the carrier after IV injection and was subsequently released slowly over a longer period. A similar interpretation has been reported for other SUN nanocarriers [58].

A more pronounced difference was observed in the liver concentration–time profile (Figure 7B). Free SUN showed relatively high liver levels at the early time points, reaching 7.48 ± 2.65 µg/g at 1 h and 5.45 ± 1.99 µg/g at 3 h, but then declined sharply to 0.15 ± 0.09 µg/g at 24 h. The slightly higher early liver concentration of free SUN is likely attributable to the immediate availability of the free drug and the known extensive tissue distribution of SUN [59]. In contrast, SeNPs-Ch-SUN maintained significantly higher liver concentrations over time, decreasing more gradually from 6.22 ± 1.03 µg/g at 1 h to 1.94 ± 0.14 µg/g at 24 h, and exhibited more sustained hepatic retention compared to free SUN. The observed hepatic distribution pattern is consistent with the physicochemical characteristics of SeNPs-Ch-SUN. The PS of 94 nm places SeNPs-Ch-SUN within a range compatible with hepatic uptake and retention, while the cationic charge of chitosan may favor interaction with negatively charged biological interfaces. In addition, the sustained-release behavior of the formulation likely contributed to the prolonged liver concentrations observed at later time points. This interpretation is also supported by previous in vivo studies showing that chitosan NPs can enhance delivery and biological activity in reticuloendothelial organs, particularly the liver and spleen [60,61].

This difference was confirmed by the exposure data summarized in Figure 7C. The plasma *AUC*_0–24_ of free SUN (42.62 ± 14.79 µg/mL·h) was higher than that of SeNPs-Ch-SUN (31.39 ± 8.45 µg/mL·h), although this difference was not statistically significant. In contrast, the liver *AUC*_0–24_ of SeNPs-Ch-SUN (77.23 ± 10.56 µg/g·h) was significantly higher than that of free SUN (36.39 ± 9.66 µg/g·h), representing approximately a 2.1-fold increase in hepatic exposure and indicating significantly improved liver distribution of SUN after administration in form on SeNPs-Ch.

To further clarify the time-dependent distribution pattern, early exposure and late retention were additionally analyzed (Supplementary Figure S3). During the early phase, AUC_0–12_ was not significantly different between free SUN and SeNPs-Ch-SUN in plasma, whereas liver AUC_0–12_ was significantly higher for SeNPs-Ch-SUN. At 24 h, SeNPs-Ch-SUN maintained significantly higher SUN concentrations in both plasma and liver, with the difference being more pronounced in liver tissue. These findings support that SeNPs-Ch-SUN did not simply increase systemic exposure, but shifted SUN disposition toward sustained hepatic retention.

The absence of a significant difference in total plasma AUC_0–24_ despite the significant increase in liver AUC_0–24_ suggests a redistribution effect rather than a generalized increase in circulating SUN exposure. This may be explained by preferential hepatic retention of the nanosized, chitosan-stabilized formulation, together with sustained release of SUN after hepatic localization. Therefore, the comparable plasma exposure and significantly higher liver exposure collectively indicate improved liver-directed distribution of SUN following administration as SeNPs-Ch-SUN.

The calculated hepatic distribution indices further supported this interpretation. The *Re* of SeNPs-Ch-SUN was 2.27 ± 0.86, indicating that the nanoparticle formulation increased liver exposure by more than two-fold relative to free SUN. Likewise, the *Te* was substantially higher for SeNPs-Ch-SUN (2.52 ± 0.33) than for free SUN (0.87 ± 0.08), and the resulting *RTe* was 2.92 ± 0.52. Collectively, these findings demonstrate that SeNPs-Ch-SUN shifted SUN disposition toward the liver to a significantly greater extent than the free drug.

These in vivo findings are consistent with previous biodistribution studies showing that SeNP-based systems can exhibit substantial hepatic accumulation after systemic administration. Singh et al. reported that indocyanine-green-conjugated SeNPs displayed preferential accumulation in the liver, followed by the testis and kidney, in a rat biodistribution study [62]. Similarly, Wang et al. demonstrated that intravenously administered Se@BSA nanoparticles were mainly detected in the kidneys and liver, as confirmed by both fluorescence imaging and ICP-MS analysis [63].

## 3. Materials and Methods

### 3.1. Materials

Sunitinib malate (purity ≥ 98%) was purchased from Sigma-Aldrich (Sigma-Aldrich, St. Louis, MO, USA). Sodium selenite (purity ≥ 99%) was purchased from LANXESS AG (Kennedy Platz 1, Cologne, Germany). Chitosan (MWT 100,000–300,000) was purchased from Across Organics (Fair Lawn, NJ, USA). Analytical-grade ascorbic acid (purity ≥ 99.5%), potassium dihydrogen phosphate (purity ≥ 99%), sodium hydroxide (purity ≥ 97%), and disodium hydrogen phosphate (pharmaceutical grade) (purity ≥ 99%) were purchased from El Nasr, Cairo, Egypt.

### 3.2. Preparation of SeNPs-Ch-SUN

SeNPs-Ch-SUN were fabricated according to a previously reported method with slight modification [64,65]. Briefly, three stock solutions were prepared: sodium selenite (50 mM) in deionized water, chitosan (1% *w*/*v*) prepared by dissolving 50 mg of chitosan in 5 mL of 2% *v*/*v* acetic acid, and ascorbic acid (100 mM) in deionized water. SUN (12.5 mg) was dissolved in 2 mL of deionized water and mixed with 2 mL of sodium selenite solution. Thereafter, 1 mL of Ch solution was added under continuous magnetic stirring to obtain a homogeneous reaction mixture. Freshly prepared ascorbic acid solution (1 mL, 100 mM) was then added dropwise under magnetic stirring (500–600 rpm) at room temperature (25 ± 2 °C). The final volume was adjusted to 10 mL with deionized water, and stirring continued for 1 h until the dispersion developed a uniform orange-red color, indicating the formation of NPs. The resulting colloidal dispersion was used as the SeNPs-Ch-SUN formulation for subsequent characterization. Blank SeNPs-Ch were prepared in the same manner in the absence of SUN.

### 3.3. Characterization of SeNPs-Ch-SUN

#### 3.3.1. Size Distribution and Zeta Potential

The mean particle size (PS), PDI, and ZP of SeNPs-Ch-SUN were determined using a Zetasizer Nano ZS instrument (Malvern Instruments, Worcestershire, UK). Size distribution was measured by dynamic light scattering (DLS), whereas ZP was determined by electrophoretic light scattering. Freshly prepared nanodispersions were appropriately diluted with filtered deionized water prior to measurement to avoid multiple scattering and to ensure the count rate remained within the instrument’s recommended operating range. All measurements were performed in triplicate (*n* = 3) at 25 °C, and results are expressed as mean ± SD.

#### 3.3.2. Loading Efficiency (LE%) Determination

The LE% of SeNPs-Ch-SUN was determined indirectly by measuring the amount of free drug remaining in the aqueous phase after ultrafiltration. Briefly, an aliquot of the freshly prepared SeNPs-Ch-SUN dispersion was transferred to an ultrafiltration tube (Amicon Ultra, molecular weight cut-off 3 kDa, Millipore, Burlington, MA, USA) and centrifuged at 5000 rpm for 10 min using a refrigerated centrifuge (Sigma 2-16P, Sigma Laborzentrifugen GmbH, Osterode am Harz, Germany). The filtrate containing the free, non-associated SUN was collected and analyzed spectrophotometrically at 267 nm using a UV–Vis spectrophotometer (UV-1900i, Shimadzu Corp. Kyoto, Japan). Blank SeNPs-Ch formulations were processed under the same conditions and used as baseline controls to correct for any carrier-related absorbance. A calibration curve prepared in water over the concentration range of 10–100 µg/mL showed excellent linearity (*R*^2^ = 0.9995). The method showed acceptable accuracy, with a mean recovery of 101 ± 1.05%, and acceptable precision, with intra-day and inter-day RSD values < 2%. All measurements were performed in triplicate (*n* = 3) at 25 °C, and results are expressed as mean ± SD. The LE% was determined using the following equation:

$$LE\;\left(\%\right)=\frac{W_{Initial}-W_{free}}{W_{Initial}}\times 100$$

#### 3.3.3. Fourier-Transform Infrared Spectroscopy (FTIR)

The FTIR spectra of SUN, chitosan, sodium selenite, ascorbic acid, their physical mixture, and lyophilized SeNPs-Ch-SUN were recorded using an FTIR spectrophotometer (BRUKER IFS 66, Bruker, Karlsruhe, Germany) over the range of 4000–400 cm^−1^ by the KBr pellet method.

#### 3.3.4. Transmission Electron Microscopy (TEM)

The morphology of SeNPs-Ch-SUN was examined by TEM using a JEM-2100 instrument (JEOL, Tokyo, Japan). A drop of the diluted nanoparticle dispersion was placed onto a carbon-coated copper grid, negatively stained with 1% phosphotungstic acid, air-dried at room temperature, and then analyzed. For quantitative size analysis, the average diameters of 100 randomly selected nanoparticles were measured from TEM images using ImageJ (NIH), and a particle-size distribution histogram was constructed. The mean TEM particle diameter was expressed as mean ± SD, *n* = 100.

#### 3.3.5. In Vitro Release and Release Kinetics

The in vitro release profile of sunitinib from SeNPs-Ch-SUN and free SUN suspension was evaluated using the dynamic dialysis bag method. Briefly, samples equivalent to 10 mg SUN were placed in dialysis bags (molecular weight cut-off 11,325 Da; DO655, Sigma-Aldrich, St. Louis, MO, USA), sealed, and immersed in 50 mL of phosphate buffer (10 mM, pH 7.4) containing 1% Tween-80 as the release medium. The system was maintained at 37 ± 0.5 °C under continuous stirring at 100 rpm. At predetermined time intervals (1, 2, 3, 4, 5, 6, 12, and 24 h), 1 mL samples were withdrawn and replaced with an equal volume of fresh medium. Blank SeNPs-Ch nanoparticles (drug-free formulation) were used as a reference to correct for any potential background interference arising from the nanoparticle system. This ensured that the measured absorbance corresponded exclusively to SUN, thereby improving the accuracy of the release quantification. The amount of SUN released was determined spectrophotometrically at 267 nm, and the cumulative percentage of drug released was calculated. For release kinetic analysis, the DDsolver software Excel based Add-In Program, version 1.0, was used [66]. The release data were fitted to zero-order, first-order, Higuchi, and Korsmeyer–Peppas models. The model with the highest correlation coefficient (*R*^2^) was considered the best fit to describe the release behavior of SUN from the developed nanosystem.

#### 3.3.6. Stability Study

The physical stability of SeNPs-Ch-SUN was evaluated over 3 months according to the general principles of the ICH Q1A(R2) guideline for stability testing of new drug substances and products, with adaptation for an aqueous nanoparticle dispersion [67]. SeNPs-Ch-SUN was evaluated over 3 months under two storage conditions: refrigerated (4 ± 2 °C) and room temperature (25 ± 2 °C). Freshly prepared formulations were transferred to tightly closed glass containers and stored under the specified conditions throughout the study period. At the specified sampling intervals (0, 1, 2, and 3 months), samples were collected from each container, and changes in PS, PDI, ZP, and LE% were measured. Each measured property at each time point was compared with the freshly prepared sample (initial values) to assess the stability of the formulation under each storage condition. All measurements were performed in triplicate, and the results were expressed as mean ± SD.

### 3.4. In Vitro Cytotoxicity

#### 3.4.1. Cell Culture and Development of Resistant HCC Cell Models

To evaluate the in vitro cytotoxicity of SeNPs-Ch-SUN compared with free SUN, parental and resistant HCC cell models were used. HepG2 cells obtained from the ATCC were cultured in RPMI-1640 medium supplemented with 10% (*v*/*v*) FBS, penicillin G (100 U/mL), and streptomycin (100 µg/mL). THLE-2 normal human hepatocytes (ATCC CRL-2706) were maintained in BEGM medium (Lonza/Clonetics, BEGM BulletKit) supplemented with 10% fetal bovine serum, additional epidermal growth factor (5 ng/mL), and phosphoethanolamine (70 ng/mL). Huh-7 cells were cultured in DMEM supplemented with 10% (*v*/*v*) FBS, penicillin G (100 U/mL), and streptomycin (100 µg/mL). All cells were maintained at 37 °C in a humidified incubator containing 5% CO_2_.

Multidrug-resistant HepG2 cells (MDR-HepG2) were developed by serial passaging of parental HepG2 cells under stepwise increasing concentrations of doxorubicin, beginning at 0.1 µM and gradually escalating to 100 µM, following previously reported multidrug-resistance selection procedures [68,69]. Cells that maintained stable proliferation under prolonged doxorubicin exposure were considered to exhibit an MDR phenotype and were used as a transporter-associated multidrug-resistant HCC model [70].

SUN-resistant Huh-7 cells were established using a previously reported method [47]. Huh-7 cells were generated by continuous exposure of parental Huh-7 cells to 5 µM SUN for 21 consecutive days. The culture medium was replaced every 3 days, and the cells were subcultured when they reached approximately 80% confluence. After the resistance-selection period, SUN-resistant Huh-7 cells were maintained in drug-free complete medium for two passages before use in cytotoxicity studies to minimize acute carryover effects while preserving the selected resistant phenotype.

#### 3.4.2. Cytotoxicity Assay

The cytotoxic activity of free SUN, blank SeNPs-Ch, and SeNPs-Ch-SUN was evaluated in parental HepG2 and Huh-7 cells, as well as in MDR-HepG2, SUN-resistant Huh-7, and THLE-2 cells, using the MTT assay. Cells were seeded in 96-well plates at a density of 1.5 × 10^4^ cells/well in 100 µL of complete culture medium and allowed to attach overnight under standard incubation conditions. The cells were then exposed to different concentrations (1.25, 2.5, 5, 10, and 20 µM) of free SUN, Blank NPs or SeNPs-Ch-SUN for 48 h. Vehicle control wells received 0.1% DMSO (*v*/*v*), matching the highest final solvent concentration in the free SUN-treated wells. Following treatment, 10 µL of MTT solution (5 mg/mL in PBS) was added to each well, and the plates were incubated for an additional 3 h at 37 °C to allow the formation of formazan crystals. Thereafter, the medium was carefully removed, and the resulting formazan crystals were dissolved in DMSO. Absorbance was measured at 570 nm with a microplate reader, and cell viability was reported as a percentage relative to untreated control cells. Measurements were performed in triplicate. In parallel, cell-free wells containing culture medium and the corresponding concentrations of blank SeNPs-Ch or SeNPs-Ch-SUN were processed under the same assay conditions and served as background controls to account for any formulation-related absorbance interference.

#### 3.4.3. Cytotoxicity Parameters

Dose–response curves were constructed by plotting the percentage of viable cells against the logarithm of concentration. The half-maximal inhibitory concentration (IC_50_) values of free SUN and SeNPs-Ch-SUN were determined by nonlinear regression analysis using GraphPad Prism version 9 software. To evaluate the effect of the formulation on resistance-associated loss of sensitivity, the resistance index (*RI*) for free SUN and SeNPs-Ch-SUN in the corresponding parental and resistant cell models was calculated separately according to the following equation [71]:

$$RI=\frac{{IC}_{50,\;resistant}}{{IC}_{50,\;parental}}$$

To assess cytotoxic selectivity toward malignant hepatic cells, the selectivity index (*SI*) was calculated for HepG2, Huh-7, MDR-HepG2, and SUN-resistant Huh-7 for both free SUN and SeNPs-Ch-SUN using THLE-2 cells as the non-malignant hepatic reference according to the following equation [72]:

$$SI=\frac{{IC}_{50,\;THLE\;cells}}{{IC}_{50,\;Cancer\;cells}}$$

### 3.5. In Vivo

#### 3.5.1. Animals

Male Wistar rats weighing 200–210 g were used in the in vivo study. The animals were housed under standard laboratory conditions for at least two weeks before the experiment, at a controlled temperature of 21 ± 2 °C and a relative humidity of 40–50%, with free access to a standard laboratory diet and water. Prior to dosing, the animals were fasted overnight, with water available ad libitum. All experimental procedures were carried out in accordance with international guidelines for the care and use of laboratory animals and were approved by the Research Ethics Committee of the Faculty of Pharmacy at Delta University under protocol number (FPDU 42/2026).

#### 3.5.2. Hepatic Distribution

To evaluate liver-targeting efficiency, rats were randomly assigned to two groups (*n* = 15/group). The first group received free SUN dissolved in deionized water, whereas the second group received SeNPs-Ch-SUN dispersed in deionized water. Both formulations were administered as a single intravenous dose of 5 mg/kg SUN at an injection volume of 1 mL/kg. At predetermined time points (1, 3, 6, 12, and 24 h) after administration, three rats from each group were sampled. Blood samples were collected from the lateral tail vein into heparinized tubes and centrifuged at 3000 rpm for 15 min to obtain plasma. The separated plasma samples were stored at −80 °C until analysis. After blood collection, rats were euthanized by cervical dislocation and immediately dissected. The liver was excised, washed with normal saline, gently blotted dry with filter paper, and accurately weighed. Liver samples were then homogenized in two volumes (*w*/*v*) of ice-cold PBS using an Ultra-Turrax homogenizer (IKA-Werke GmbH & Co. KG, Staufen im Breisgau, Germany). The final volume of each liver homogenate was recorded, and the samples were stored at −80 °C until assay.

SUN concentration–time profiles in plasma and liver were plotted for both formulations. The *AUC*_0–24_ was calculated using the PKSolver software Excel based Add-In Program, version 2.0 [73]. To compare the hepatic distribution of SeNPs-Ch-SUN with free SUN, the following targeting indices were determined: relative intake rate (*Re*), targeting efficiency (*Te*), and relative targeting efficiency (*RTe*) derived from tissue and plasma AUC values according to the following equations:

$$Re=\;\frac{{AUC}_{0-24\;Liver,\;SeNPs-Ch-SUN\;}}{{AUC}_{0-24\;Liver,\;Free\;SUN}}$$

$$Te=\frac{{AUC}_{0-24\;Liver\;}}{{AUC}_{0-24\;Plasma}}$$

$$RTe=\frac{Te\;(SeNPs-Ch-SUN)}{\;Te\;(Free\;SUN)}$$

An *Re* value greater than 1 indicates increased hepatic uptake of the nanoparticle formulation relative to free SUN, whereas an *RTe* value greater than 1 indicates improved hepatic targeting efficiency of SeNPs-Ch-SUN compared with the free drug.

#### 3.5.3. HPLC Analysis

SUN analysis in plasma and liver homogenates was performed using HPLC method. A 0.25 mL aliquot of either plasma or liver homogenate was mixed with 0.25 mL of 0.1 N sodium hydroxide, followed by the addition of 2.5 mL ethyl acetate as the extracting solvent. The mixture was vortexed thoroughly and centrifuged for 10 min at 4000 rpm at room temperature. The resulting supernatant was filtered through a 0.22 μm membrane filter and evaporated to dryness in a water bath at 40 °C. The residue was reconstituted in 200 μL of the mobile phase, and a 50 μL aliquot of the solution was injected into the HPLC system for analysis.

The HPLC system comprised a Shimadzu CBM-20 A Lite controller, an SPD-20 A UV-visible detector set at 431 nm, an LC-20 AT pump, a CTO-20 A column oven maintained at 40 °C, and a DGU-20A3R degassing unit. Separation was performed on a C18 reversed-phase column (5 μm, 4.6 × 250 mm, 130 Å; Waters, Milford, MA, USA). The mobile phase consisted of acetonitrile and 20 mM ammonium acetate buffer (pH 6.8) in a ratio of 55:45 (*v*/*v*), delivered isocratically at a flow rate of 1 mL/min throughout the run. Data acquisition and processing were conducted using LabSolutions LC/GC software (version 5.84, Shimadzu, Kyoto, Japan). Under these chromatographic conditions, SUN showed a retention time of approximately 5.3 min. The method exhibited excellent linearity over the concentration range of 50–500 ng/mL, with correlation coefficients (R^2^) of 0.9998 and 0.9996 for plasma and liver homogenates, respectively. High recovery values were obtained for both matrices, reaching 99.05 ± 1.04% for plasma and 97.13 ± 0.95% for liver homogenates, while intra- and inter-day precision was satisfactory with %RSD values below 2%, confirming the accuracy and reproducibility of the assay.

### 3.6. Statistical Analysis

Statistical analysis was performed using GraphPad Prism software (version 9.0; GraphPad Software, San Diego, CA, USA). Comparisons between two groups were carried out using Student’s *t*-test, whereas comparisons among more than two groups were performed by analysis of variance (ANOVA) followed by post hoc multiple-comparison test. A *p* value < 0.05 was considered statistically significant. For cytotoxicity studies, dose–response curves were analyzed by nonlinear regression to determine the IC_50_ values. All experiments were conducted in triplicate, and the data are presented as mean ± SD.

## 4. Conclusions

The present study successfully developed SeNPs-Ch as an effective nanocarrier for SUN delivery to the liver. The prepared formulation displayed favorable physicochemical characteristics, including nanoscale PS (93.62 ± 1.06 nm), a narrow size distribution (PDI = 0.205 ± 0.01), a positive surface charge (+24.47 ± 1.31 mV), high loading efficiency (83.8 ± 2.16%), and a spherical, non-aggregated morphology, while FTIR findings supported the successful association of SUN with the SeNPs-Ch. The formulation also demonstrated sustained drug release, with 51.17 ± 1.26% of SUN released after 24 h compared with almost complete release of free SUN within 3 h. Release-kinetic analysis showed the best fit to the Korsmeyer–Peppas model, with an exponent value of *n* = 0.198 ± 0.042, indicating diffusion-dominated release. Most importantly, SeNPs-Ch-SUN improved the biological performance of SUN across the conducted studies. In vitro, the nanoformulation enhanced cytotoxicity against both parental and resistant HCC cell models, improved selectivity toward malignant cells over normal hepatocytes, and reduced multidrug-resistance-associated loss of sensitivity in MDR-HepG2 cells. In vivo, SeNPs-Ch-SUN increased hepatic exposure after iv administration, with liver AUC_0–24_ increasing from 36.39 ± 9.66 µg/g·h for free SUN to 77.23 ± 10.56 µg/g·h for SeNPs-Ch-SUN, corresponding to an approximately 2.1-fold increase in liver exposure. The formulation also produced favorable hepatic-distribution indices, including a relative intake rate of 2.27 ± 0.86 and relative targeting efficiency of 2.92 ± 0.52. These findings support the ability of SeNPs-Ch-SUN to improve SUN hepatic exposure and enhance its in vitro anticancer performance in HCC-relevant models.

The novelty of this work lies in engineering SeNPs-Ch-SUN as a liver-directed selenium–chitosan nanoparticle platform that integrates the carrier-related biological activity of selenium, the stabilizing and cell-interactive properties of chitosan, and the anticancer activity of SUN into a single nanosystem for HCC-oriented delivery. Nonetheless, these results should be interpreted in light of the study’s limitations. Because the in vivo study was performed in normal rats rather than HCC-bearing rats, the data demonstrate increased hepatic distribution and retention but do not prove selective targeting of HCC tumor tissue. The resistant-cell results indicate enhanced intracellular delivery and partial escape from drug resistance in the MDR study; however, the exact mechanisms underlying these effects (e.g., uptake into cells and mechanisms that circumvent drug resistance) were beyond the scope of the current study. Thus, future studies should examine the utility of SeNPs-Ch-SUN in orthotopic or xenograft HCC model systems, compare the intratumoral and extratumoral distributions of the compound, determine the mechanism by which SeNPs-Ch-SUN modulates cellular uptake and efflux-related mechanisms of drug resistance, and investigate the long-term efficacy and safety of repeated administration. In conclusion, SeNPs-Ch-SUN supports the hypothesis that this formulation can act as a liver-targeting nanocarrier to improve the accumulation profile and anticancer effect of SUN in HCC treatment.

## Figures and Tables

**Table 1:** Comparative cytotoxicity, resistance indices, and selectivity profiles of free SUN and SeNPs-Ch-SUN in normal and HCC cell models after 48 h exposure. Data are expressed as mean ± SD, *n* = 3.

Cell Model	IC_50_ (µM)		RI		SI	
	Free SUN	SeNPs-Ch-SUN	Free SUN	SeNPs-Ch-SUN	Free SUN	SeNPs-Ch-SUN
THLE-2	13.56 ± 2.69	>20 *	—	—	—	—
Parental Huh-7	4.75 ± 0.707	2.61 ± 0.575	—	—	2.85 ± 0.14	>7.91
SUN-resistant Huh-7	12.74 ± 1.81	7.12 ± 0.959	2.68 ± 0.01	2.76 ± 0.24	1.06 ± 0.05	>2.84
Parental HepG2	3.31 ± 0.56	2.01 ± 0.485	—	—	4.07 ± 0.11	>10.42
MDR-HepG2	17.55 ± 3.09	6.78 ± 1.070	5.29 ± 0.13	3.44 ± 0.46	0.77 ± 0.02	>2.99

For SeNPs-Ch-SUN in THLE-2 cells, the IC_50_ was not reached within the tested concentration range; therefore, * the value is expressed as >20 µM and the corresponding SI values are presented as minimum estimates. Blank SeNPs-Ch did not reach 50% inhibition within the tested concentration range; therefore, IC_50_ values were not calculated for the blank formulation.

## Data Availability

The original contributions presented in this study are included in the article. Further inquiries can be directed to the corresponding authors.

## Supplementary Materials

The following supporting information can be downloaded at: https://www.mdpi.com/article/10.3390/ph19060898/s1, Figure S1: Visual appearance of the prepared SeNPs-Ch-SUN colloidal dispersion. The formulation appeared as a homogeneous orange-red colloidal dispersion, with no visible macroscopic phase separation or precipitation; Figure S2: Pairwise statistical comparison of in vitro cytotoxicity among free SUN, SeNPs-Ch-SUN, and blank SeNPs-Ch after 48 h exposure. Cell viability was assessed in THLE-2 normal hepatocytes, parental Huh-7 cells, SUN-resistant Huh-7 cells, parental HepG2 cells, and MDR-HepG2 cells at matched concentrations. Data are presented as mean ± SD (n = 3). Statistical comparisons were performed among treatment groups at each concentration. Significance is indicated as follows: ns, non-significant; * *p* < 0.05; ** *p* < 0.01; *** *p* < 0.001; **** *p* < 0.0001; Figure S3: Early exposure and late retention of SUN after intravenous administration of free SUN and SeNPs-Ch-SUN. (A) Early exposure expressed as AUC_0–12_ in plasma and liver. Plasma AUC is expressed as µg/mL·h, whereas liver AUC is expressed as µg/g·h. (B) SUN concentration at 24 h in plasma and liver, showing late systemic and hepatic retention. Data are presented as mean ± SD (n = 3). Statistical comparisons were performed between free SUN and SeNPs-Ch-SUN within each matrix. Significance is indicated as follows: ns, non-significant; ** *p* < 0.01; **** *p* < 0.0001; Table S1: Major FTIR spectral changes observed for SUN, excipients, physical mixture, and SeNPs-Ch-SUN.

## References

[1] Konyn P., Ahmed A., Kim D. (2021). Current epidemiology in hepatocellular carcinoma. Expert Rev. Gastroenterol. Hepatol..

[2] Anand N., Wu S., Guo Z., Figueroa M.S., Jung L., Li M., Bhan I., Franses J.W. (2025). Novel Blood-Based Liquid Biopsy Approaches in Hepatocellular Carcinoma. JCO Oncol. Adv..

[3] Li P., Ding Z., Feng Y., Ren X., Wei Y., Xia C., Yang Y., Yang Q., Wang Z., Zhang X. (2026). Global, regional, and national burden of hepatocellular carcinoma and contribution of nine modifiable risk factors across 185 countries/territories in 2022. Sci. Bull..

[4] Kim D.Y. (2024). Changing etiology and epidemiology of hepatocellular carcinoma: Asia and worldwide. J. Liver Cancer.

[5] Singh S.P., Madke T., Chand P. (2025). Global Epidemiology of Hepatocellular Carcinoma. J. Clin. Exp. Hepatol..

[6] Leowattana W., Leowattana T., Leowattana P. (2023). Systemic treatment for unresectable hepatocellular carcinoma. World J. Gastroenterol..

[7] Zou Y., Wan X., Zhou Q., Zhu G., Lin S., Tang Q., Yang X., Wang S. (2025). Mechanisms of drug resistance in hepatocellular carcinoma. Biol. Proced. Online.

[8] Marin J.J.G., Macias R.I.R., Monte M.J., Romero M.R., Asensio M., Sanchez-Martin A., Cives-Losada C., Temprano A.G., Espinosa-Escudero R., Reviejo M. (2020). Molecular Bases of Drug Resistance in Hepatocellular Carcinoma. Cancers.

[9] Qi D., Qin Y., Zhu H., Li Y., Han S. (2025). Resistance of first-line targeted drugs in hepatocellular carcinoma: The epigenetic regulation mechanisms. Cell Death Dis..

[10] Llovet J.M., Pinyol R., Kelley R.K., El-Khoueiry A., Reeves H.L., Wang X.W., Gores G.J., Villanueva A. (2022). Molecular pathogenesis and systemic therapies for hepatocellular carcinoma. Nat. Cancer.

[11] Donne R., Lujambio A. (2023). The liver cancer immune microenvironment: Therapeutic implications for hepatocellular carcinoma. Hepatology.

[12] Papaetis G.S., Syrigos K.N. (2009). Sunitinib: A multitargeted receptor tyrosine kinase inhibitor in the era of molecular cancer therapies. BioDrugs.

[13] Christensen J.G. (2007). A preclinical review of sunitinib, a multitargeted receptor tyrosine kinase inhibitor with anti-angiogenic and antitumour activities. Ann. Oncol..

[14] Majumder S., Piguet A.C., Dufour J.F., Chatterjee S. (2013). Study of the cellular mechanism of Sunitinib mediated inactivation of activated hepatic stellate cells and its implications in angiogenesis. Eur. J. Pharmacol..

[15] Cheng A.L., Kang Y.K., Lin D.Y., Park J.W., Kudo M., Qin S., Chung H.C., Song X., Xu J., Poggi G. (2013). Sunitinib versus sorafenib in advanced hepatocellular cancer: Results of a randomized phase III trial. J. Clin. Oncol..

[16] Faivre S., Raymond E., Boucher E., Douillard J., Lim H.Y., Kim J.S., Zappa M., Lanzalone S., Lin X., Deprimo S. (2009). Safety and efficacy of sunitinib in patients with advanced hepatocellular carcinoma: An open-label, multicentre, phase II study. Lancet Oncol..

[17] Tian T., Ruan J., Zhang J., Zhao C.X., Chen D., Shan J. (2022). Nanocarrier-Based Tumor-Targeting Drug Delivery Systems for Hepatocellular Carcinoma Treatments: Enhanced Therapeutic Efficacy and Reduced Drug Toxicity. J. Biomed. Nanotechnol..

[18] Liu Y., Wu Y., Li Z., Wan D., Pan J. (2024). Targeted Drug Delivery Strategies for the Treatment of Hepatocellular Carcinoma. Molecules.

[19] Nasr M., Kira A.Y., Saber S., Essa E.A., El-Gizawy S.A. (2023). Telmisartan-Loaded lactosylated chitosan nanoparticles as a liver specific delivery system: Synthesis, optimization and targeting efficiency. AAPS PharmSciTech.

[20] Yu Z., Guo J., Liu Y., Wang M., Liu Z., Gao Y., Huang L. (2022). Nano Delivery of Simvastatin Targets Liver Sinusoidal Endothelial Cells to Remodel Tumor Microenvironment for Hepatocellular Carcinoma. J. Nanobiotechnol..

[21] Hamad R.S., Saber S., Sameh A., Elmorsy E.A., Farrag A.A., Eissa H., El-Kott A.F., AlShehri M.A., Alfarteesh H.A., Eltantawy W. (2026). A Spatial-Mechanistic Design Map for Microenvironment-Responsive Nanomaterials in Solid Tumors. FASEB J..

[22] Vahidi H., Barabadi H., Saravanan M. (2020). Emerging selenium nanoparticles to combat cancer: A systematic review. J. Clust. Sci..

[23] Varlamova E.G., Goltyaev M.V., Mal’tseva V.N., Turovsky E.A., Sarimov R.M., Simakin A.V., Gudkov S.V. (2021). Mechanisms of the Cytotoxic Effect of Selenium Nanoparticles in Different Human Cancer Cell Lines. Int. J. Mol. Sci..

[24] Xia Y., Zhong J., Zhao M., Tang Y., Han N., Hua L., Xu T., Wang C., Zhu B. (2019). Galactose-modified selenium nanoparticles for targeted delivery of doxorubicin to hepatocellular carcinoma. Drug Deliv..

[25] Guo M., Tang Y., Hua L., Li W., Gong G., Zhu Y., Zhu B., Xia Y. (2022). Functionalized Selenium Nanoparticles Enhance Anticancer Efficacy of Doxorubicin for Hepatocellular Carcinoma Therapy. Adv. Mater. Sci. Eng..

[26] Cui F., Huang Q.X., Yu Z., Yang J.J., Liang S.Y., Wang M.Q., Zhu J., Tian C., Li S.X., Wang H.T. (2025). Biomimetic selenium nanomedicine with homologous targeting enhances hepatocellular carcinoma therapy. Mater. Today Bio.

[27] Khaled A.M., Othman M.S., Obeidat S.T., Aleid G.M., Aboelnaga S.M., Fehaid A., Hathout H.M.R., Bakkar A.A., Moneim A.E.A., El-Garawani I.M. (2024). Green-Synthesized Silver and Selenium Nanoparticles Using Berberine: A Comparative Assessment of In Vitro Anticancer Potential on Human Hepatocellular Carcinoma Cell Line (HepG2). Cells.

[28] Kira A.Y., Abdelhamid A.M., Nasr M. (2024). Navigating liver targeting: Fine-tuning chitosan nanocarriers through saccharide modification. J. Drug Deliv. Sci. Technol..

[29] Kira A.Y., Elmorsy E.A., Hamad R.S., Abdel-Reheim M.A., Elhemely M.A., El Adle Khalaf N., El-Kott A.F., AlShehri M.A., Morsy K., Negm S. (2024). Nicardipine-chitosan nanoparticles alleviate thioacetamide-induced acute liver injury by targeting NFkappaB/NLRP3/IL-1beta signaling in rats: Unraveling new roles beyond calcium channel blocking. Int. Immunopharmacol..

[30] Elmorsy E.A., Saber S., Kira A.Y., Alghasham A., Abdel-Hamed M.R., Amer M.M., Mohamed E.A., AlSalloom A.A., Alkhamiss A.S., Hamad R.S. (2024). Hedgehog signaling is a promising target for the treatment of hepatic fibrogenesis: A new management strategy using itraconazole-loaded nanoparticles. Front. Pharmacol..

[31] Nasr M., Kira A.Y., Saber S., Essa E.A., El-Gizawy S.A. (2023). Lactosylated Chitosan Nanoparticles Potentiate the Anticancer Effects of Telmisartan In Vitro and in a N-Nitrosodiethylamine-Induced Mice Model of Hepatocellular Carcinoma. Mol. Pharm..

[32] Szafranska K., Neuman T., Baster Z., Rajfur Z., Szelest O., Holte C., Kubisiak A., Kus E., Wolfson D.L., Chlopicki S. (2022). From fixed-dried to wet-fixed to live–comparative super-resolution microscopy of liver sinusoidal endothelial cell fenestrations. Nanophotonics.

[33] Zapotoczny B., Szafranska K., Lekka M., Ahluwalia B.S., McCourt P. (2022). Tuning of liver sieve: The interplay between actin and myosin regulatory light chain regulates fenestration size and number in murine liver sinusoidal endothelial cells. Int. J. Mol. Sci..

[34] Axelsson O., Yousefpour N., Bjornberg O., Ekengard E., Lekmeechai S. (2024). Size-dependent renal filtration model explains human pharmacokinetics of a functional nanoparticle: The SPAGOPIX-01 clinical trial. Nanomedicine.

[35] Kaplan A.B.U., Çetin M. (2024). Preparation and in vitro characterization of carbamazepine-loaded chitosan-coated/uncoated PLGA and Zein nanoparticles. Black Sea J. Eng. Sci..

[36] Hamad R.S., Saber S., Farrag A.A., Eissa H., Elmorsy E.A., El-Kott A.F., AlShehri M.A., Al-Shahari E.A., Eltantawy W., Ali M.A. (2026). The Nano–Biointerface as a Structural Regulator of Cell Behavior: Interactions of Extracellular Vesicles, Synthetic Nanocarriers, and Nanofibers with Cells. Tissue Cell.

[37] Kassem M.G., Motiur Rahman A.F.M., Korashy H.M., Brittain H.G. (2012). Chapter 9-Sunitinib Malate. Profiles of Drug Substances, Excipients and Related Methodology.

[38] Frost R.L., Keeffe E.C. (2008). Raman spectroscopic study of the selenite mineral mandarinoite Fe_2_Se_3_O_9_·6H_2_O. J. Raman Spectrosc..

[39] Thombare N., Mahto A., Singh D., Chowdhury A.R., Ansari M.F. (2023). Comparative FTIR Characterization of Various Natural Gums: A Criterion for Their Identification. J. Polym. Environ..

[40] Golonka I., Kizior B., Szyja B.M., Damek M.P., Musial W. (2022). Assessment of the Influence of the Selected Range of Visible Light Radiation on the Durability of the Gel with Ascorbic Acid and Its Derivative. Int. J. Mol. Sci..

[41] Joseph J.J., Sangeetha D., Gomathi T. (2016). Sunitinib loaded chitosan nanoparticles formulation and its evaluation. Int. J. Biol. Macromol..

[42] Elmorsy E.A., Saber S., Al-Majdoub Z.M., Hamad R.S., Abdel-Reheim M.A., Ramadan A., Alsoqih N.S., Alharbi M.S., Alsaykhan H., Farrag A.A. (2025). Innovative pH-responsive alginate-coated rosuvastatin-loaded chitosan nanoparticles: A targeted approach to inhibiting HMGB1-activated RAGE/TLR4-NFkappaB signaling in colonic inflammation in rats. Front. Pharmacol..

[43] Elmorsy E.A., Saber S., Alsoqih N.S., Alsaykhan H., Alsalloom A.A., Hamza E., Hamad R.S., Almansour Z.H., Khodeir M.M., El-kott A.F. (2025). Targeted intranasal brain delivery of brilliant blue G via mucoadhesive spanlastic nanovesicles attenuates neuroinflammation by limiting cGAS–STING signaling. J. Drug Deliv. Sci. Technol..

[44] Saber M.M., Bahrainian S., Dinarvand R., Atyabi F. (2017). Targeted drug delivery of Sunitinib Malate to tumor blood vessels by cRGD-chiotosan-gold nanoparticles. Int. J. Pharm..

[45] Xia Y., Xiao M., Zhao M., Xu T., Guo M., Wang C., Li Y., Zhu B., Liu H. (2020). Doxorubicin-loaded functionalized selenium nanoparticles for enhanced antitumor efficacy in cervical carcinoma therapy. Mater. Sci. Eng. C Mater. Biol. Appl..

[46] Din F.U., Aman W., Ullah I., Qureshi O.S., Mustapha O., Shafique S., Zeb A. (2017). Effective use of nanocarriers as drug delivery systems for the treatment of selected tumors. Int. J. Nanomed..

[47] Dehghan S., Naghipour A., Anbaji F.Z., Golshanrad P., Mirazi H., Adelnia H., Bodaghi M., Far B.F. (2023). Enhanced in vitro and in vivo anticancer activity through the development of Sunitinib-Loaded nanoniosomes with controlled release and improved uptake. Int. J. Pharm..

[48] Da Silva C.G., Peters G.J., Ossendorp F., Cruz L.J. (2017). The potential of multi-compound nanoparticles to bypass drug resistance in cancer. Cancer Chemother. Pharmacol..

[49] Kirtane A.R., Kalscheuer S.M., Panyam J. (2013). Exploiting nanotechnology to overcome tumor drug resistance: Challenges and opportunities. Adv. Drug Deliv. Rev..

[50] Ashrafizadeh M., Hushmandi K., Mirzaei S., Bokaie S., Bigham A., Makvandi P., Rabiee N., Thakur V.K., Kumar A.P., Sharifi E. (2023). Chitosan-based nanoscale systems for doxorubicin delivery: Exploring biomedical application in cancer therapy. Bioeng. Transl. Med..

[51] Imran H., Tang Y., Wang S., Yan X., Liu C., Guo L., Wang E., Xu C. (2023). Optimized DOX Drug Deliveries via Chitosan-Mediated Nanoparticles and Stimuli Responses in Cancer Chemotherapy: A Review. Molecules.

[52] Guo L., Gong H., Tang T.-L., Zhang B.-K., Zhang L.-Y., Yan M. (2021). Crizotinib and Sunitinib Induce Hepatotoxicity and Mitochondrial Apoptosis in L02 Cells via ROS and Nrf2 Signaling Pathway. Front. Pharmacol..

[53] Tang T.L., Yang Y., Guo L., Xia S., Zhang B., Yan M. (2022). Sunitinib induced hepatotoxicity in L02 cells via ROS-MAPKs signaling pathway. Front. Pharmacol..

[54] Song X., Chen Y., Zhao G., Sun H., Che H., Leng X. (2020). Effect of molecular weight of chitosan and its oligosaccharides on antitumor activities of chitosan-selenium nanoparticles. Carbohydr. Polym..

[55] Estevez H., Garcia-Lidon J.C., Luque-Garcia J.L., Camara C. (2014). Effects of chitosan-stabilized selenium nanoparticles on cell proliferation, apoptosis and cell cycle pattern in HepG2 cells: Comparison with other selenospecies. Colloids Surf. B Biointerfaces.

[56] Sun S.-J., Deng P., Peng C.-E., Ji H.-Y., Mao L.-F., Peng L.-Z. (2022). Selenium-modified chitosan induces HepG2 cell apoptosis and differential protein analysis. Cancer Manag. Res..

[57] Yongvongsoontorn N., Chung J.E., Gao S.J., Bae K.H., Yamashita A., Tan M.-H., Ying J.Y., Kurisawa M. (2019). Carrier-enhanced anticancer efficacy of sunitinib-loaded green tea-based micellar nanocomplex beyond tumor-targeted delivery. ACS Nano.

[58] Yueh P.F., Chiang C.S., Tsai I.J., Tseng Y.L., Chen H.R., Lan K.L., Hsu F.T. (2024). A multifunctional PEGylated liposomal-encapsulated sunitinib enhancing autophagy, immunomodulation, and safety in renal cell carcinoma. J. Nanobiotechnol..

[59] Gerritse S.L., Labots M., Ter Heine R., Dekker H., Poel D., Tauriello D.V.F., Nagtegaal I.D., Van Den Hombergh E., Van Erp N., Verheul H.M.W. (2022). High-Dose Intermittent Treatment with the Multikinase Inhibitor Sunitinib Leads to High Intra-Tumor Drug Exposure in Patients with Advanced Solid Tumors. Cancers.

[60] Anand N., Kanwar R.K., Sehgal R., Kanwar J.R. (2016). Antiparasitic and immunomodulatory potential of oral nanocapsules encapsulated lactoferrin protein against *Plasmodium berghei*. Nanomedicine.

[61] Anand N., Sehgal R., Kanwar R.K., Dubey M.L., Vasishta R.K., Kanwar J.R. (2015). Oral administration of encapsulated bovine lactoferrin protein nanocapsules against intracellular parasite *Toxoplasma gondii*. Int. J. Nanomed..

[62] Singh H., Kaur J., Datusalia A.K., Naqvi S. (2023). Age-dependent assessment of selenium nanoparticles: Biodistribution and toxicity study in young and adult rats. Nanomedicine.

[63] Wang S., Chen Y., Han S., Liu Y., Gao J., Huang Y., Sun W., Wang J., Wang C., Zhao J. (2022). Selenium nanoparticles alleviate ischemia reperfusion injury-induced acute kidney injury by modulating GPx-1/NLRP3/Caspase-1 pathway. Theranostics.

[64] Srag El-Din A.S.G., Yehia A., Hamza E., A-Elgadir T.M.E., Abd El-Fattah E.E. (2024). Selenium nanoparticle ameliorates LPS-induced acute lung injury in rats through inhibition of ferroptosis, inflammation, and HSPs. J. Drug Deliv. Sci. Technol..

[65] Ramadan A., Hamza E., Elkordy E.A., Abd El Fattah E.E., Yehia A., El-Din A.S.G.S. (2026). Mechanistic Modulation of Lipopolysaccharide-Induced Hepatic Injury by Chitosan-Coated Selenium Nanoparticles: Targeting the STEAP-3/TLR-4 and IL-17/TRAF-6/HSP-90 Axes. Pharmaceutics.

[66] Saber S., Nasr M., Yahya G., Elagamy H.I., Zaid M.H.A., Sharaf H., Kira A.Y. (2025). Silk fibroin/gelatin electrospun nanofibrous dressing loaded with roxadustat accelerates wound healing in diabetic rats via HIF-1α stabilization. J. Drug Deliv. Sci. Technol..

[67] ICH (2003). ICH Harmonised Tripartite Guideline: Stability Testing of New Drug Substances and Products Q1A(R2); Current Step 4 Version.

[68] Madrid M.F., Mendoza E.N., Padilla A.L., Choquenaira-Quispe C., de Jesus Guimaraes C., de Melo Pereira J.V., Barros-Nepomuceno F.W.A., Lopes Dos Santos I., Pessoa C., de Moraes Filho M.O. (2025). In vitro models to evaluate multidrug resistance in cancer cells: Biochemical and morphological techniques and pharmacological strategies. J. Toxicol. Environ. Health B Crit. Rev..

[69] Pummarin S., Madared N., Kayem S., Orzechowski S., Teesalu T., Moya S., Boonla C. (2024). Different doxorubicin sensitivity across various human cancer cell lines. Trends Sci..

[70] Cho Y., Kim Y.K. (2020). Cancer stem cells as a potential target to overcome multidrug resistance. Front. Oncol..

[71] Yang J.X., Luo Y., Qiu H.M., Tang W.X. (2009). Characterization and resistance mechanisms of cisplatin-resistant human hepatocellular carcinoma cell line. Saudi Med. J..

[72] Lopez-Lazaro M. (2015). A simple and reliable approach for assessing anticancer activity in vitro. Curr. Med. Chem..

[73] Zhang Y., Huo M., Zhou J., Xie S. (2010). PKSolver: An add-in program for pharmacokinetic and pharmacodynamic data analysis in Microsoft Excel. Comput. Methods Programs Biomed..

