# Supplementary material for: Engineering Selenium–Chitosan Nanoparticles for Enhanced Hepatic Delivery of Sunitinib and Improved In Vitro Anticancer Activity in Hepatocellular Carcinoma Models
Source: Pharmaceuticals (Basel). 2026 Jun 5;19(6):898. doi: 10.3390/ph19060898 (PMC13304551; doi:10.3390/ph19060898)
Supplement: Supplementary file 1 [file pharmaceuticals-19-00898-s001.zip › pharmaceuticals-4328549-supplementary.pdf]

Table S1. Major FTIR spectral changes observed for SUN, excipients, physical mixture, and SeNPs-Ch-SUN.

Component: reference band	Assignment	Observation in SeNPs-Ch-SUN	Interpretation
SUN: ~3340 cm^{-1}	N–H stretching	Merged into a broad band centered near 3426 cm^{-1}	Suggests overlap with chitosan O–H/N–H groups and involvement in hydrogen-bonding interactions
SUN: ~1424 cm^{-1}	C–H bending vibration	Shifted to ~1415 cm^{-1}	Indicates the altered chemical environment of SUN after nanoparticle incorporation
SUN: ~1018 cm^{-1}	C–N stretching	Shifted to ~1000 cm^{-1} with reduced sharpness	Supports the association of SUN with the nanoparticle matrix through non-covalent interactions
Chitosan: ~3421 cm^{-1}	O–H/N–H stretching	Retained as the dominant broad band around 3426 cm^{-1}	Supports the presence of chitosan in the final nanosystem and its role in surface stabilization
Chitosan: ~1650 cm^{-1}	Amide-related vibration	Slightly shifted to ~1648 cm^{-1}	Suggests participation of chitosan functional groups in nanoparticle stabilization/intermolecular interactions
Chitosan: ~1070 cm^{-1}	C–O–C stretching of polysaccharide backbone	Present but partially overlapped	Consistent with the retention of chitosan structure in the formulated nanoparticles
Sodium selenite: ~740 cm^{-1}	O–Se–O bending vibration	Disappeared	Consistent with consumption/reduction of selenite during selenium nanoparticle formation
Ascorbic acid: ~1750, 1650, 1050 cm^{-1}	Carbonyl/C=C/C–O related vibrations	Reduced intensity and/or overlapped	Consistent with the involvement of ascorbic acid during the reduction process
Physical mixture	Retained characteristic bands of individual components	No marked band displacement	Indicates the absence of major interaction before nanoparticle formation

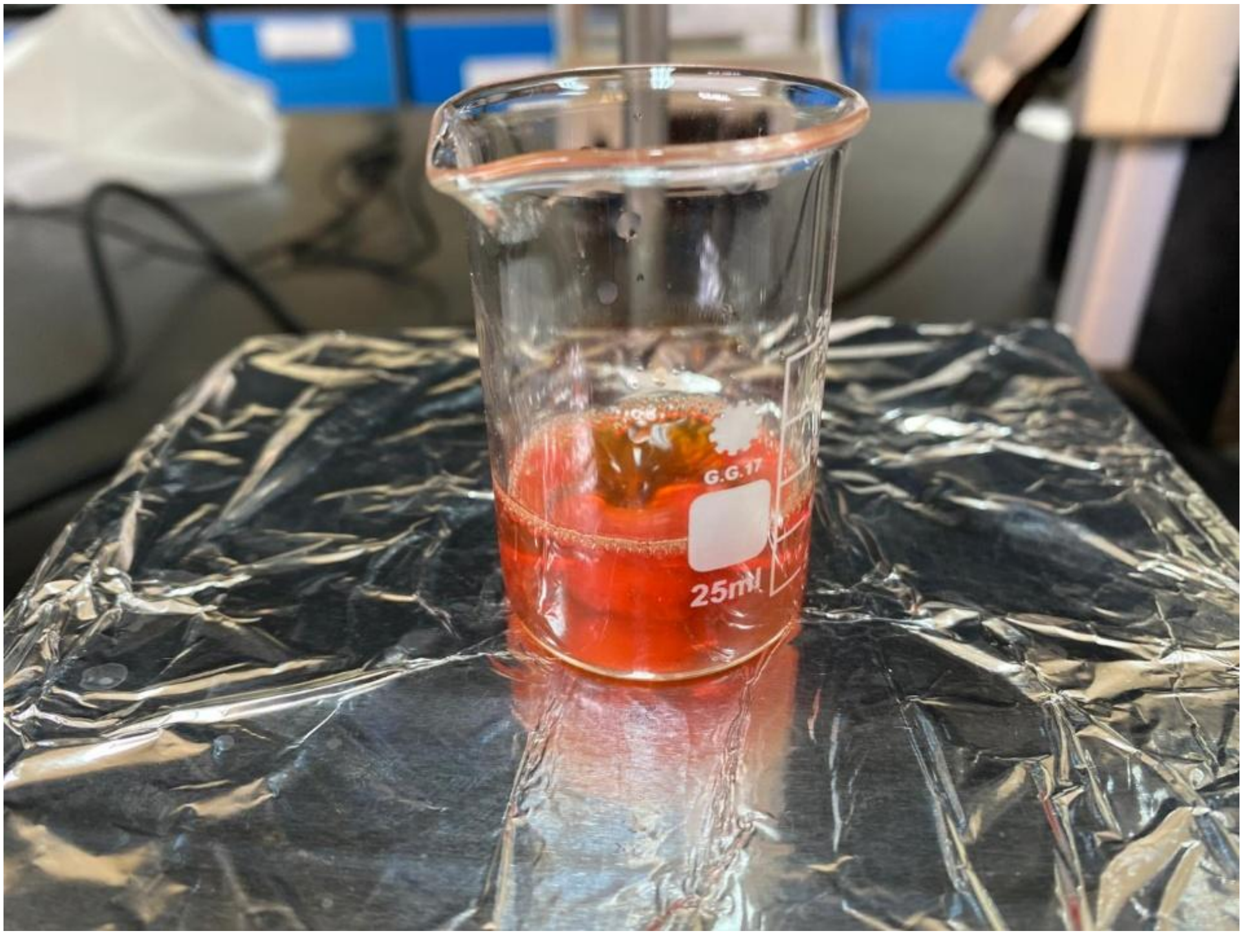

Figure S1. Visual appearance of the prepared SeNPs-Ch-SUN colloidal dispersion. The formulation appeared as a homogeneous orange-red colloidal dispersion, with no visible macroscopic phase separation or precipitation.

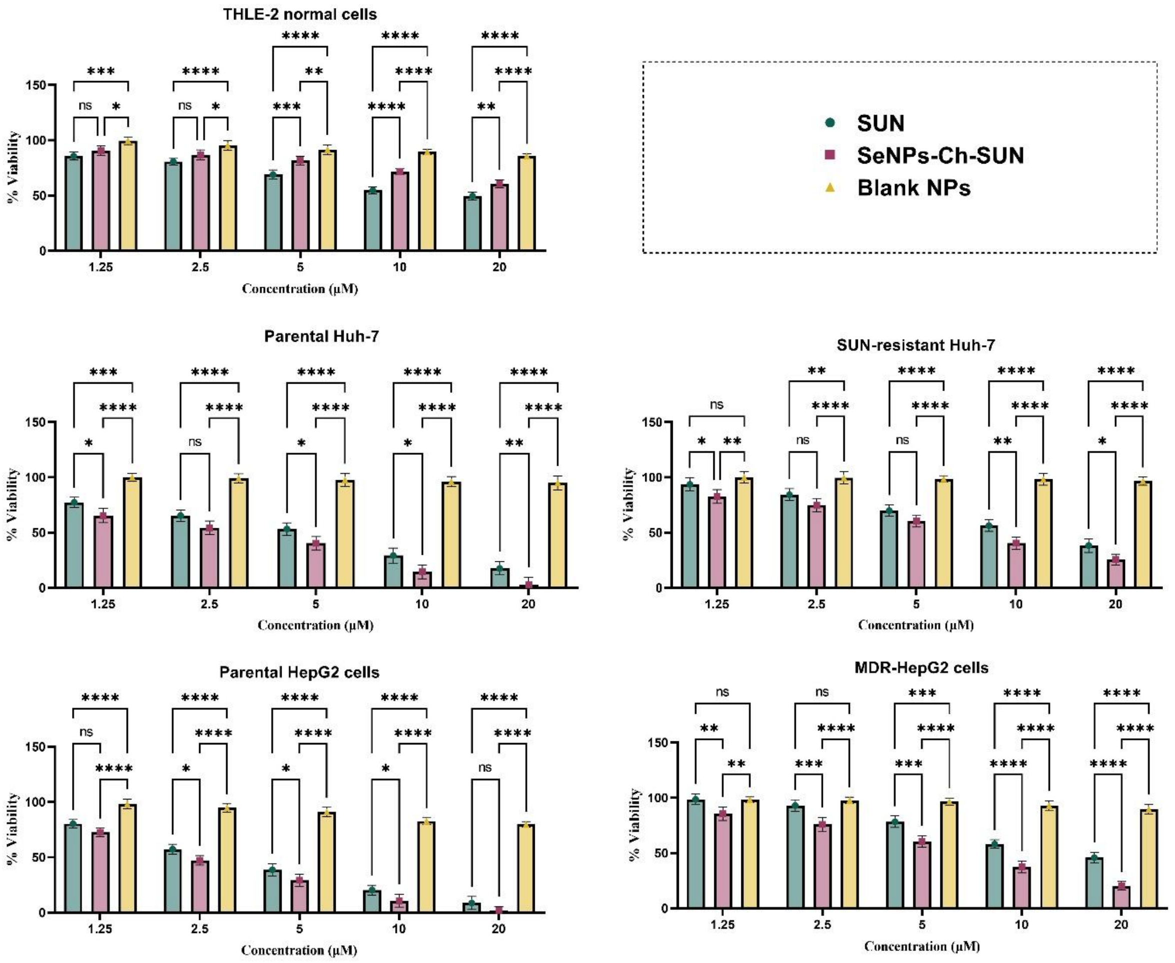

Figure S2. Pairwise statistical comparison of in vitro cytotoxicity among free SUN, SeNPs-Ch-SUN, and blank SeNPs-Ch after 48 h exposure. Cell viability was assessed in THLE-2 normal hepatocytes, parental Huh-7 cells, SUN-resistant Huh-7 cells, parental HepG2 cells, and MDR-HepG2 cells at matched concentrations. Data are presented as mean ± SD (n = 3). Statistical comparisons were performed among treatment groups at each concentration. Significance is indicated as follows: ns, non-significant; *p < 0.05; **p < 0.01; ***p < 0.001; ****p < 0.0001.

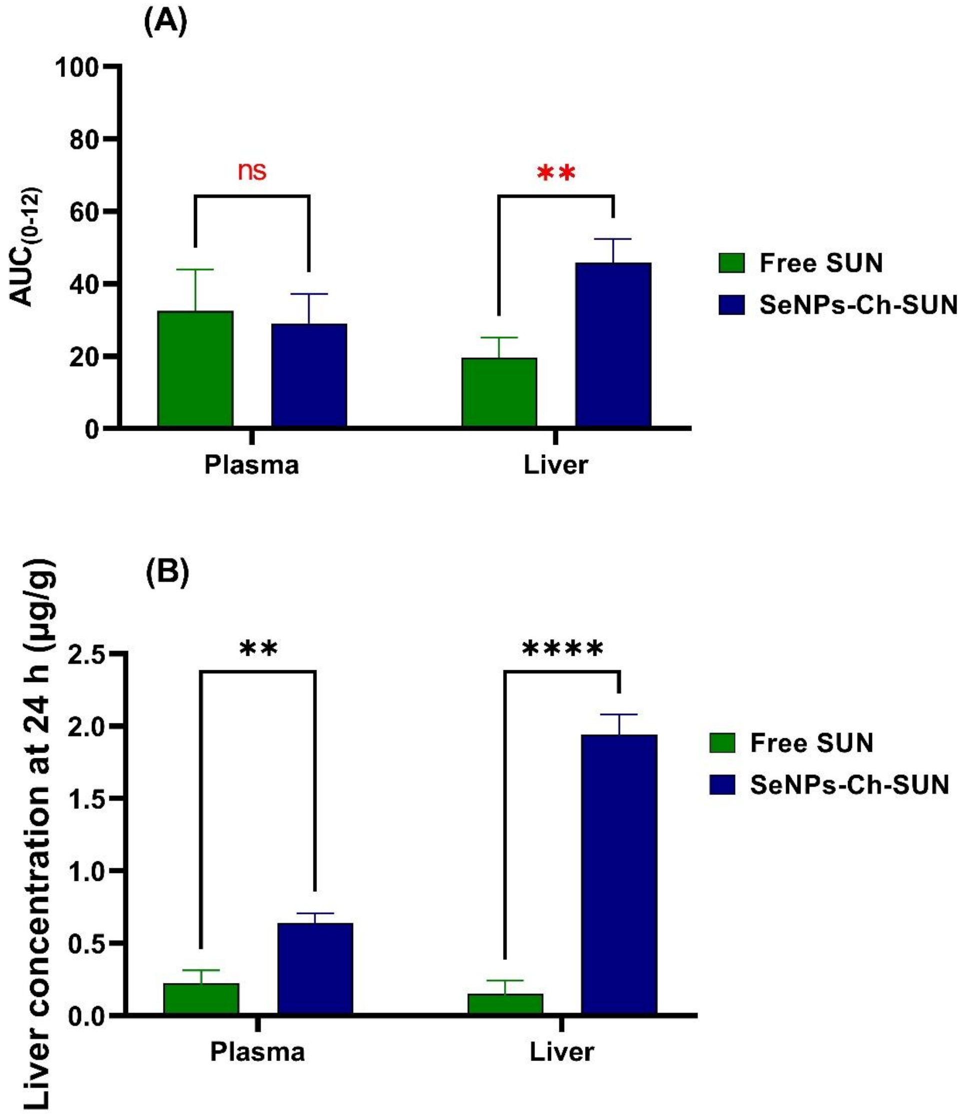

Figure S3. Early exposure and late retention of SUN after intravenous administration of free SUN and SeNPs-Ch-SUN. (A) Early exposure expressed as $AUC_{0–12}$ in plasma and liver. Plasma AUC is expressed as µg/mL·h, whereas liver AUC is expressed as µg/g·h. (B) SUN concentration at 24 h in plasma and liver, showing late systemic and hepatic retention. Data are presented as mean ± SD (n = 3). Statistical comparisons were performed between free SUN and SeNPs-Ch-SUN within each matrix. Significance is indicated as follows: ns, non-significant; $**p < 0.01$; $****p < 0.0001$.
